# Supplementary figures and images for: Bilateral regulation of EGFR activity and local PI(4,5)P2 dynamics in mammalian cells observed with superresolution microscopy
Source: eLife. 2024 Nov 8;13:e101652. doi: 10.7554/eLife.101652 (PMC11548882; doi:10.7554/eLife.101652)

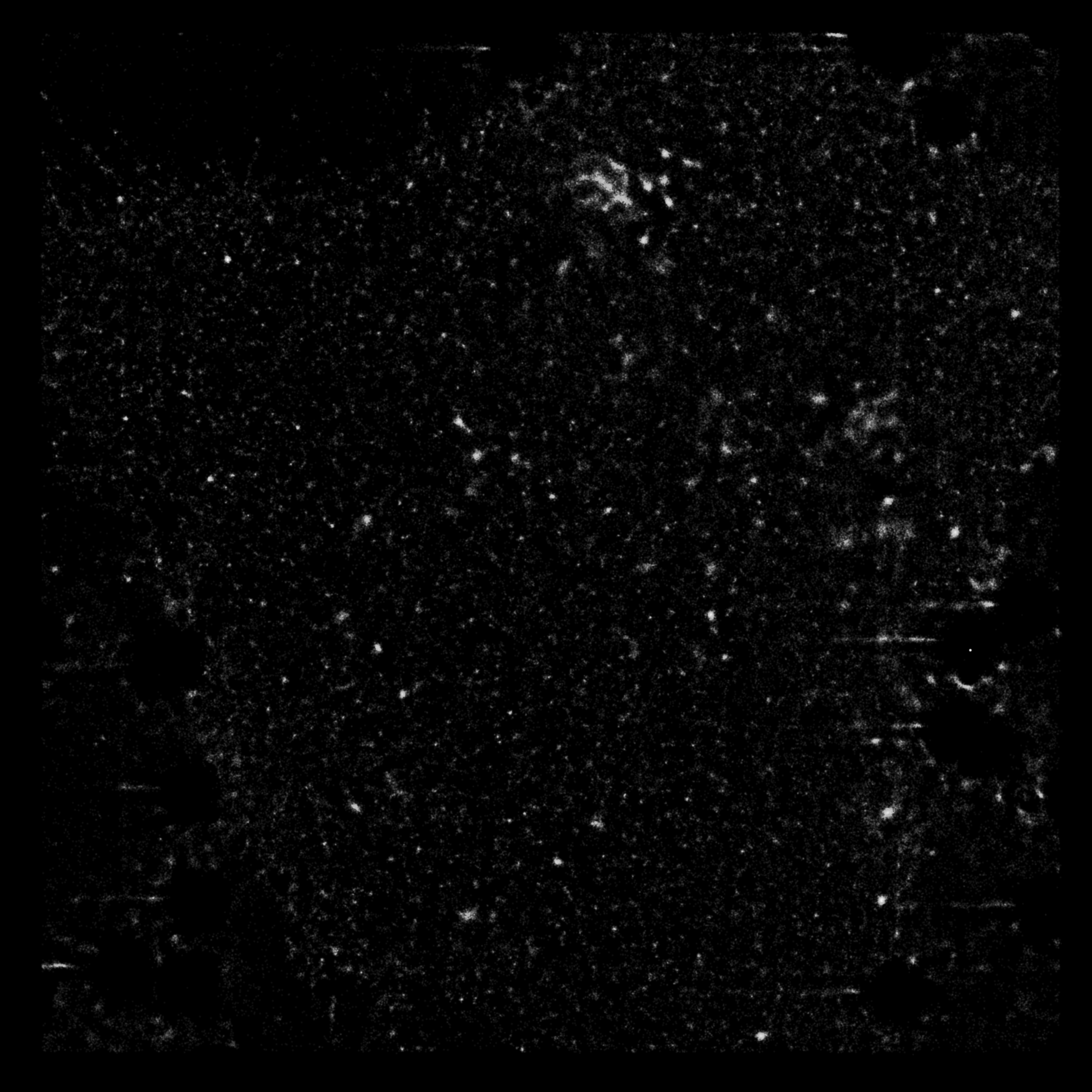

Supplement: Figure 1—source data 1. [file elife-101652-fig1-data1.zip › Figure 1-source data 1/Figure 1A-PI(4,5)P2.tif]

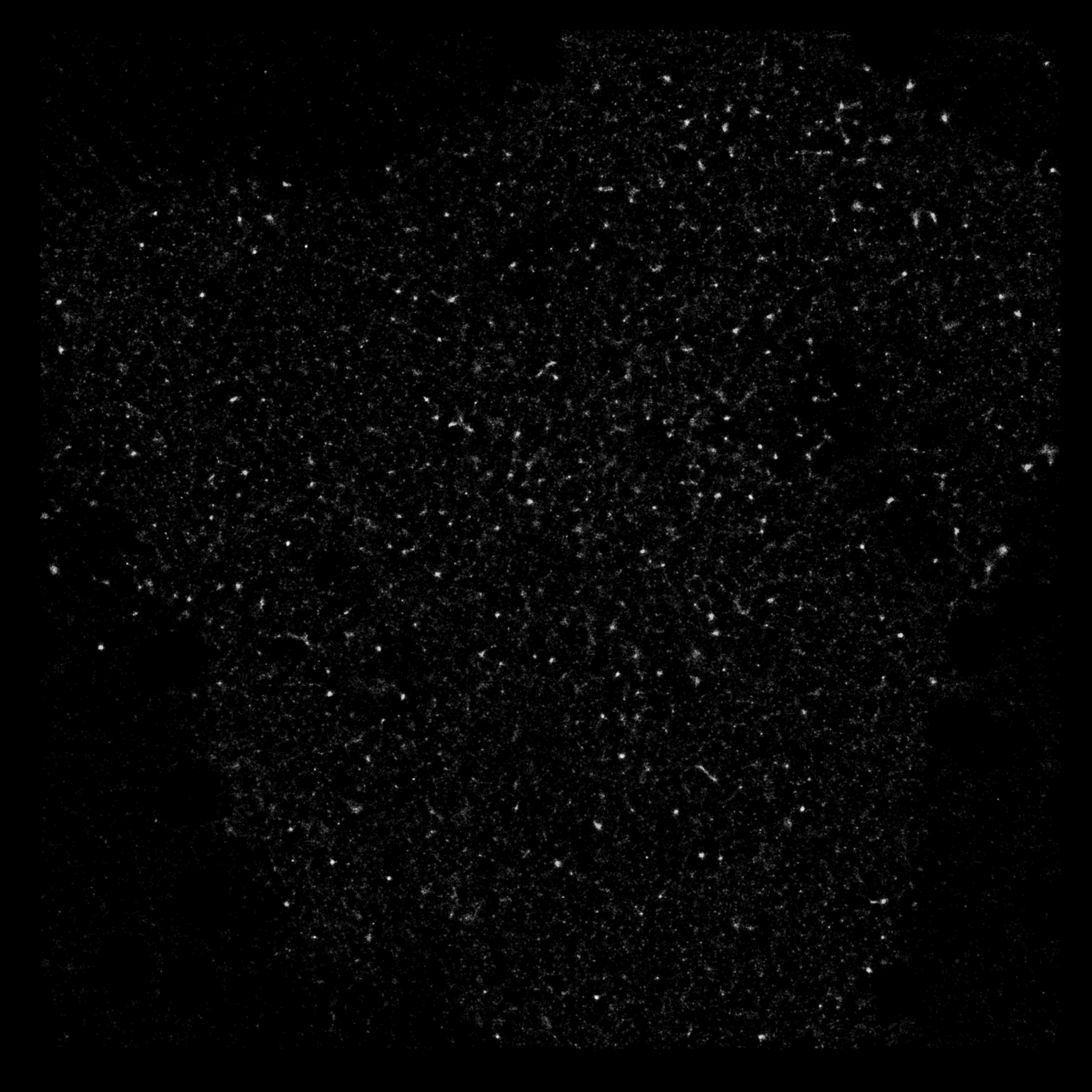

Supplement: Figure 1—source data 1. [file elife-101652-fig1-data1.zip › Figure 1-source data 1/Figure 1A-PS.tif]

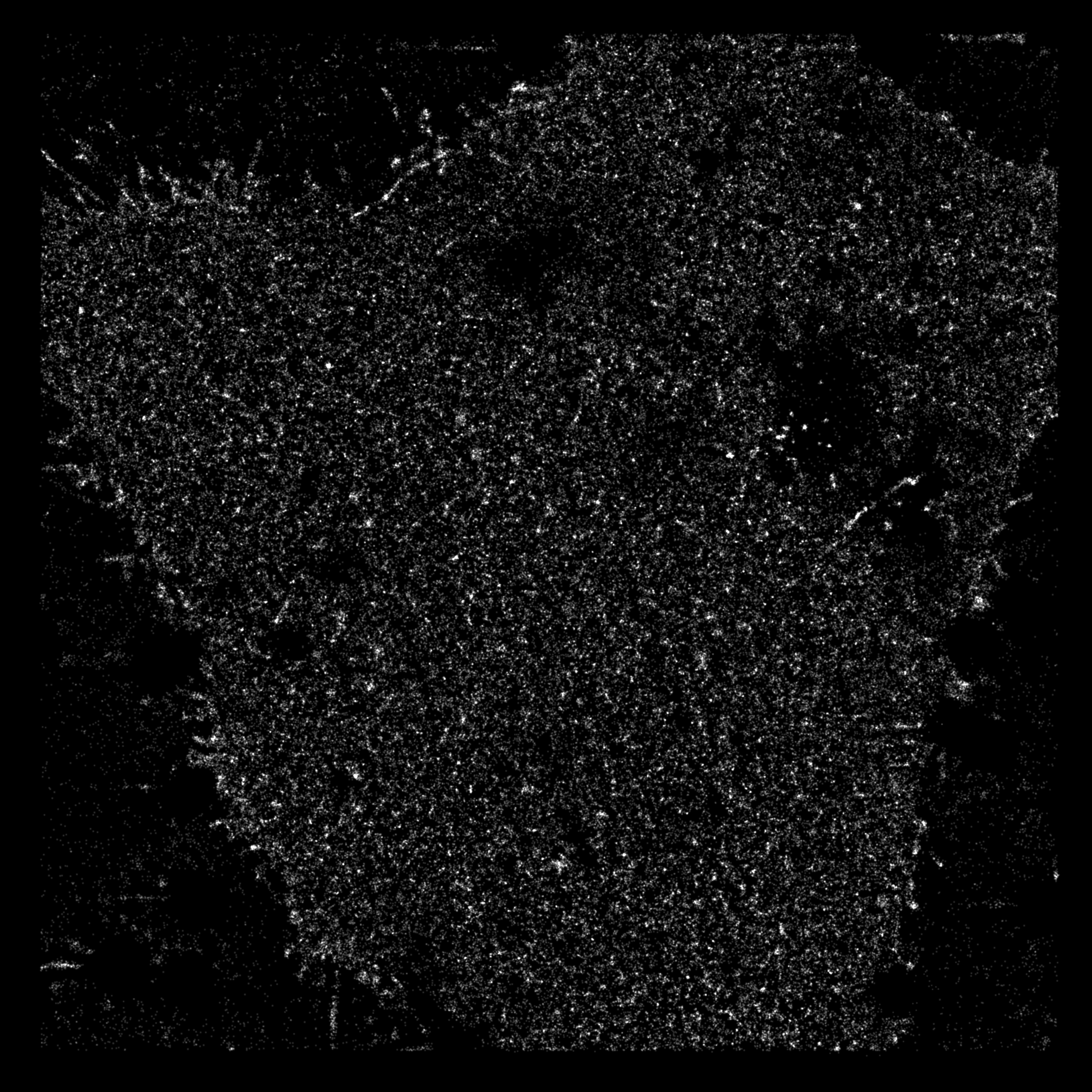

Supplement: Figure 1—source data 1. [file elife-101652-fig1-data1.zip › Figure 1-source data 1/Figure 1A-EGFR.tif]

A

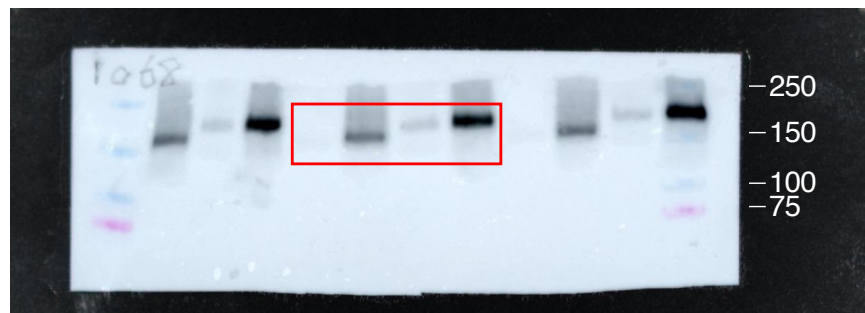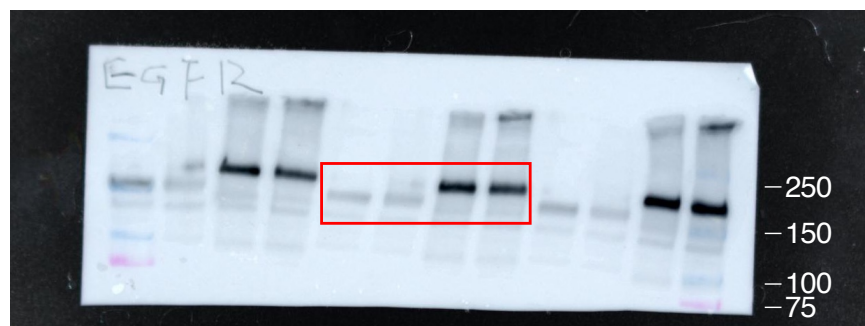

B

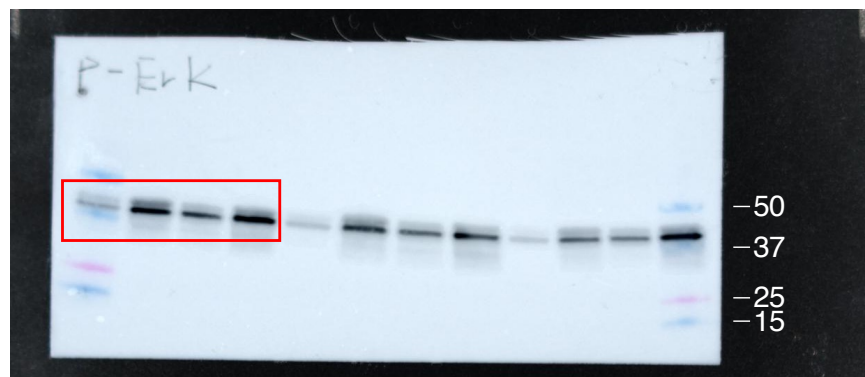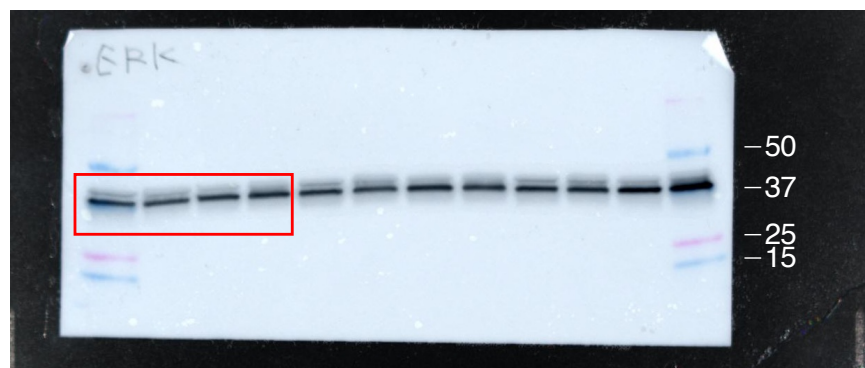

Supplement: Figure 1—figure supplement 2—source data 1. [file elife-101652-fig1-figsupp2-data1.zip › Figure 1-figure supplement 2-source data 1.pdf]

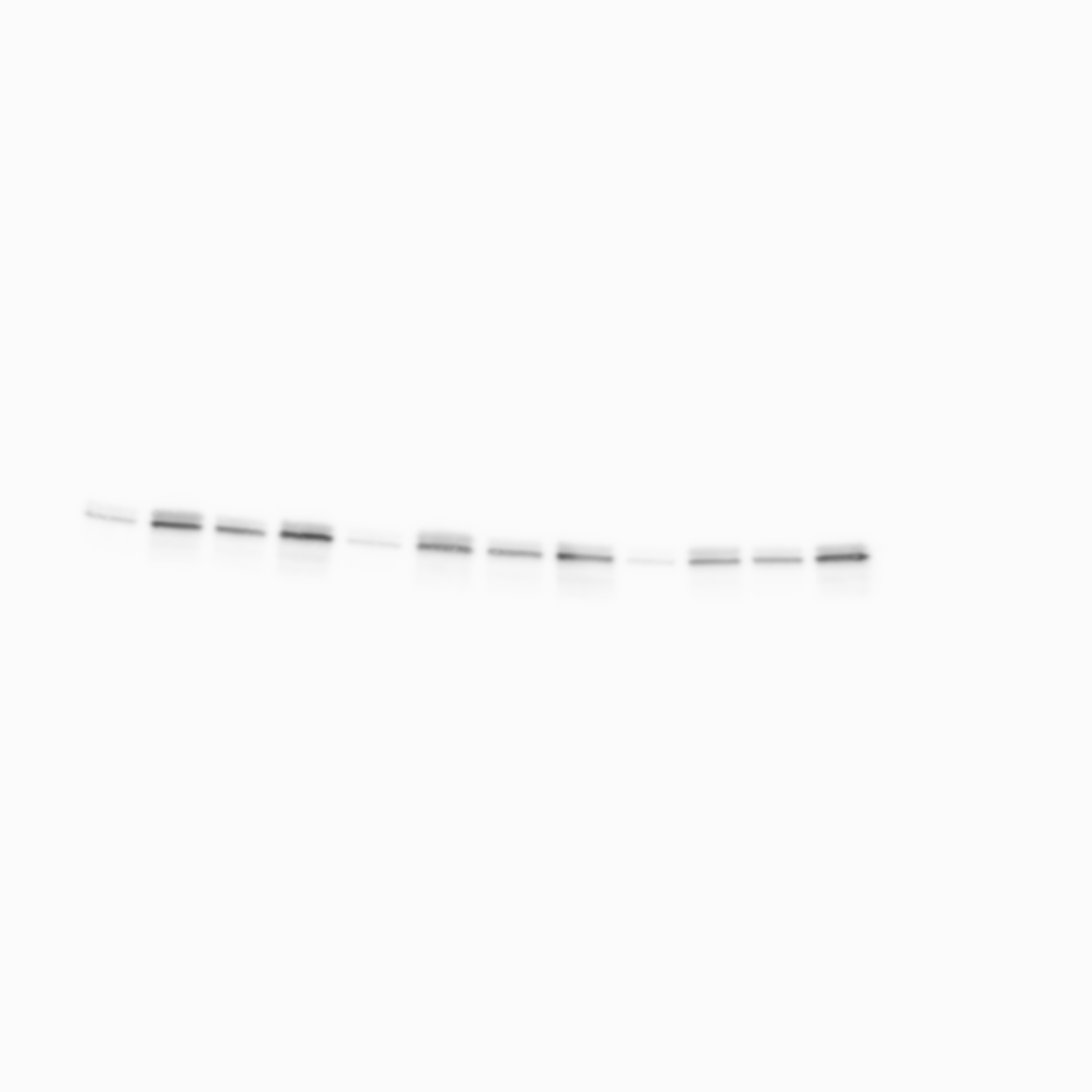

Supplement: Figure 1—figure supplement 2—source data 2. [file elife-101652-fig1-figsupp2-data2.zip › Figure 1-figure supplement 2-source data 2/Figure 1-supplement 2B-pERK.tif]

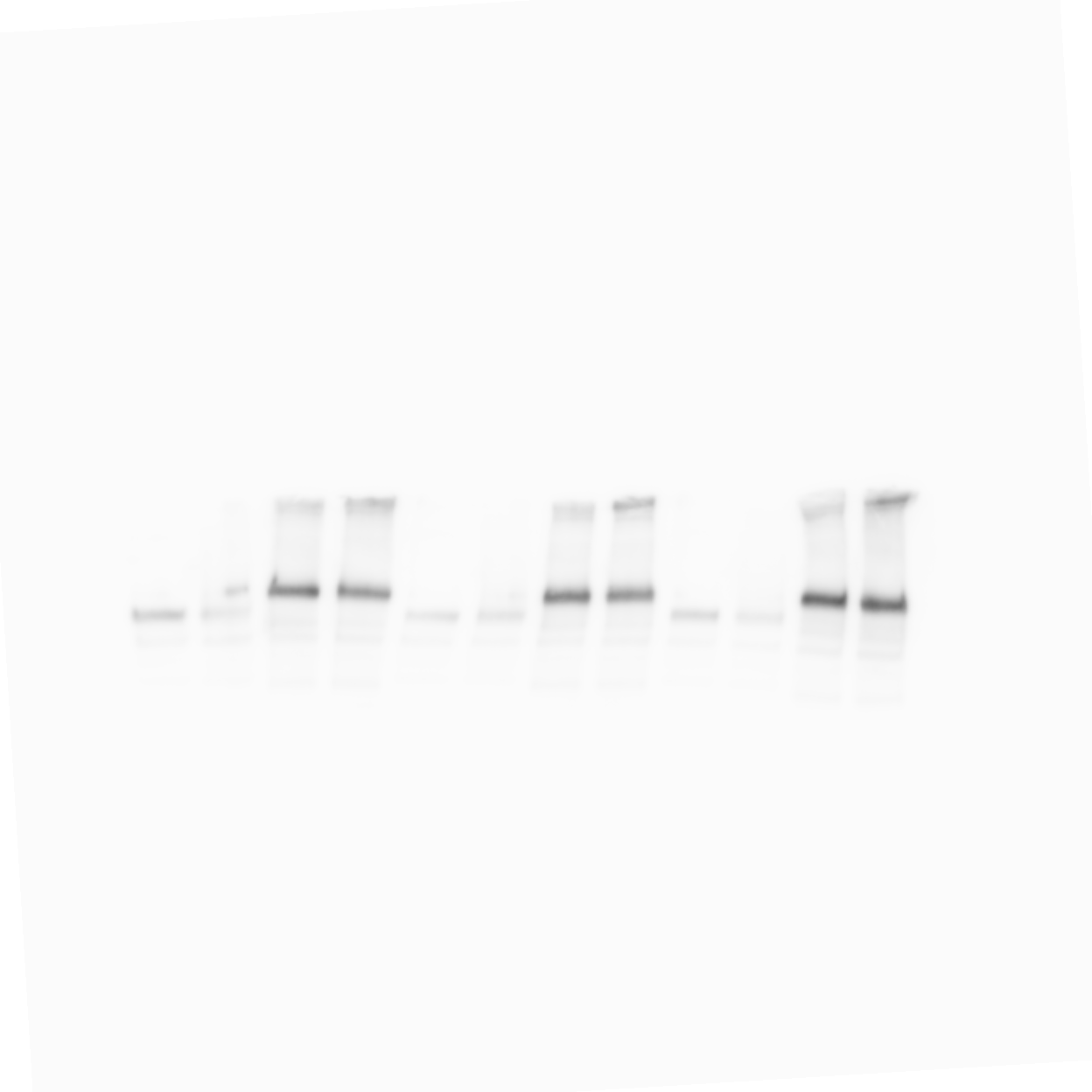

Supplement: Figure 1—figure supplement 2—source data 2. [file elife-101652-fig1-figsupp2-data2.zip › Figure 1-figure supplement 2-source data 2/Figure 1-supplement 2A-EGFR.tif]

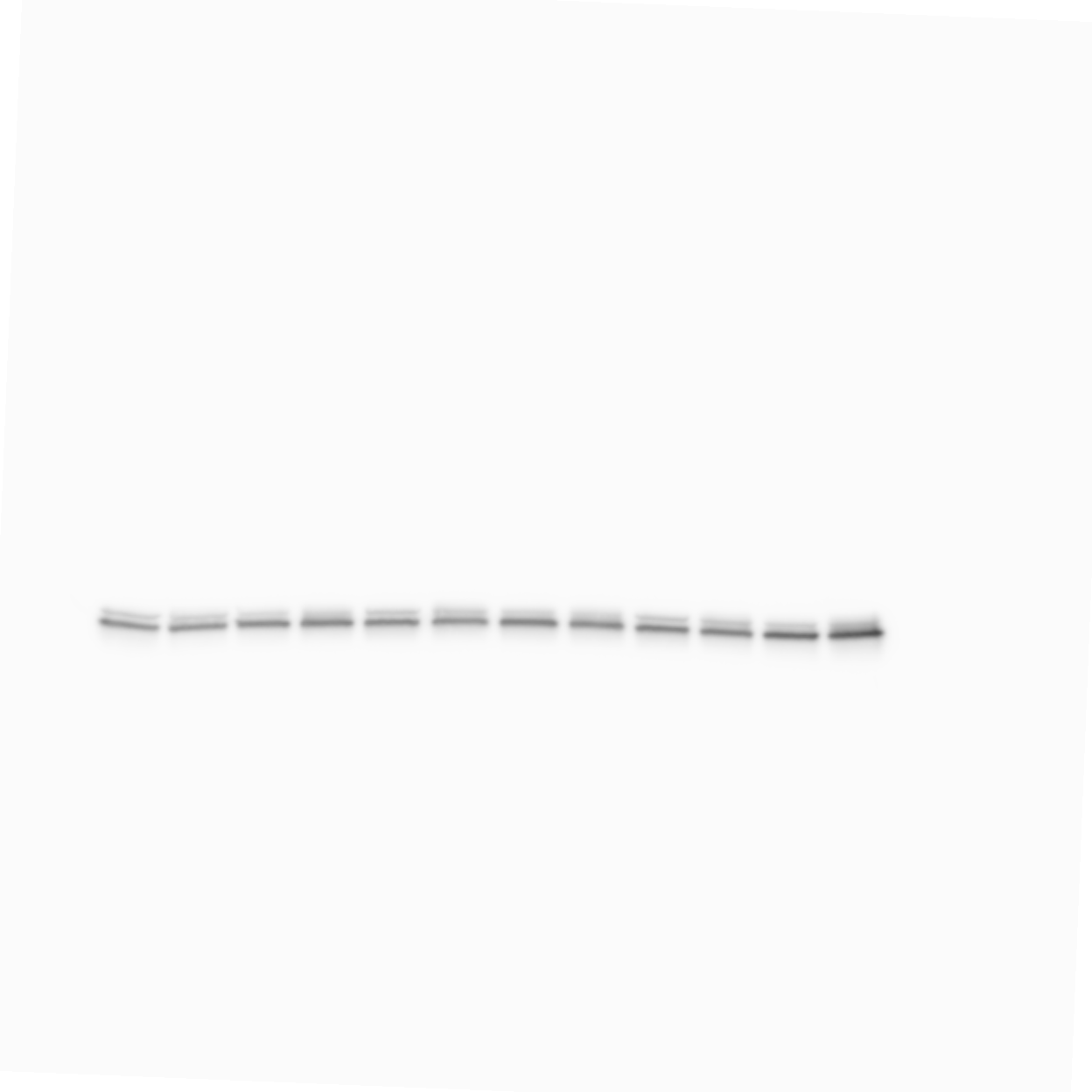

Supplement: Figure 1—figure supplement 2—source data 2. [file elife-101652-fig1-figsupp2-data2.zip › Figure 1-figure supplement 2-source data 2/Figure 1-supplement 2B-ERK.tif]

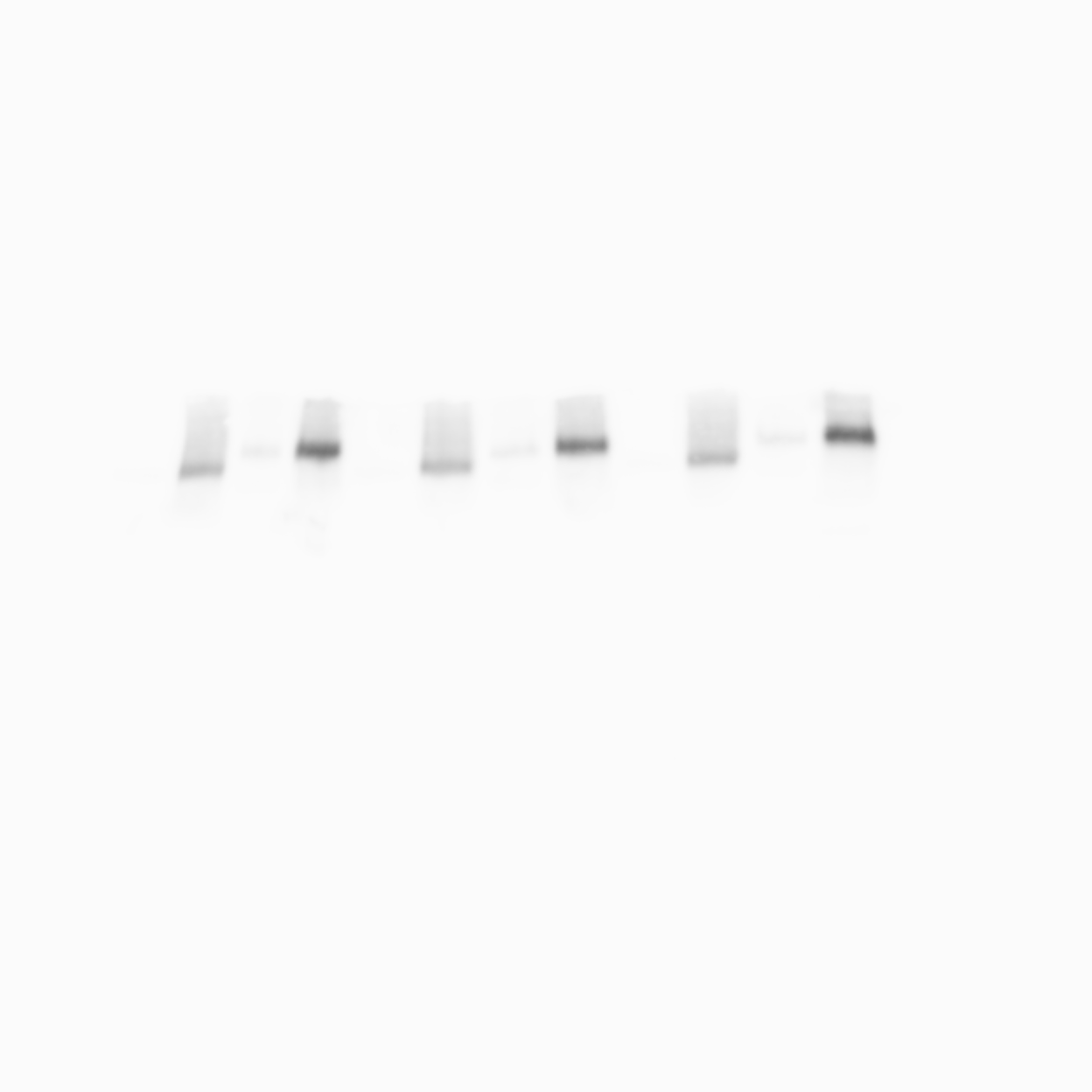

Supplement: Figure 1—figure supplement 2—source data 2. [file elife-101652-fig1-figsupp2-data2.zip › Figure 1-figure supplement 2-source data 2/Figure 1-supplement 2A-pY1068.tif]

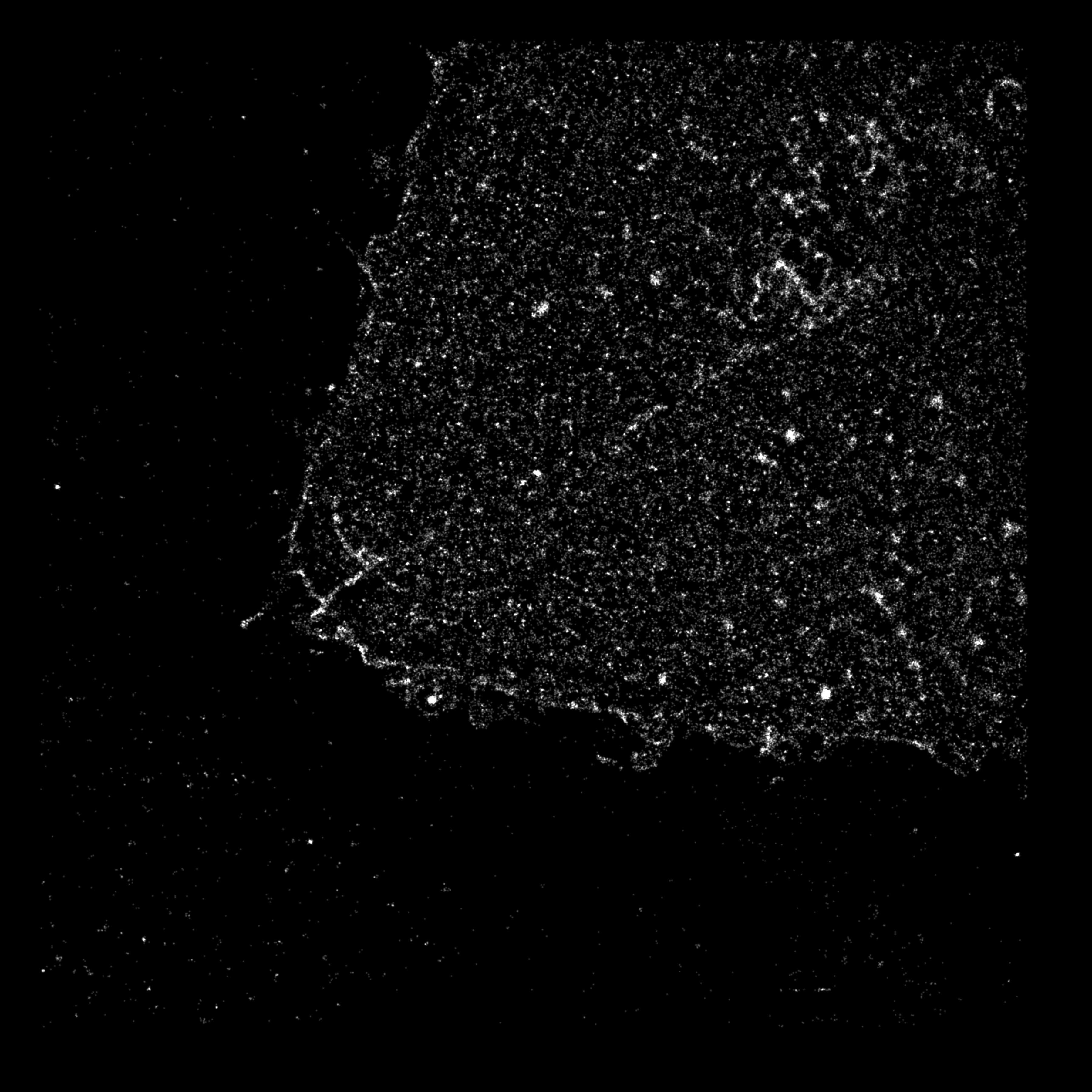

Supplement: Figure 2—source data 2. [file elife-101652-fig2-data2.zip › Figure 2-source data 2/Figure 2F-right-EGFR.tif]

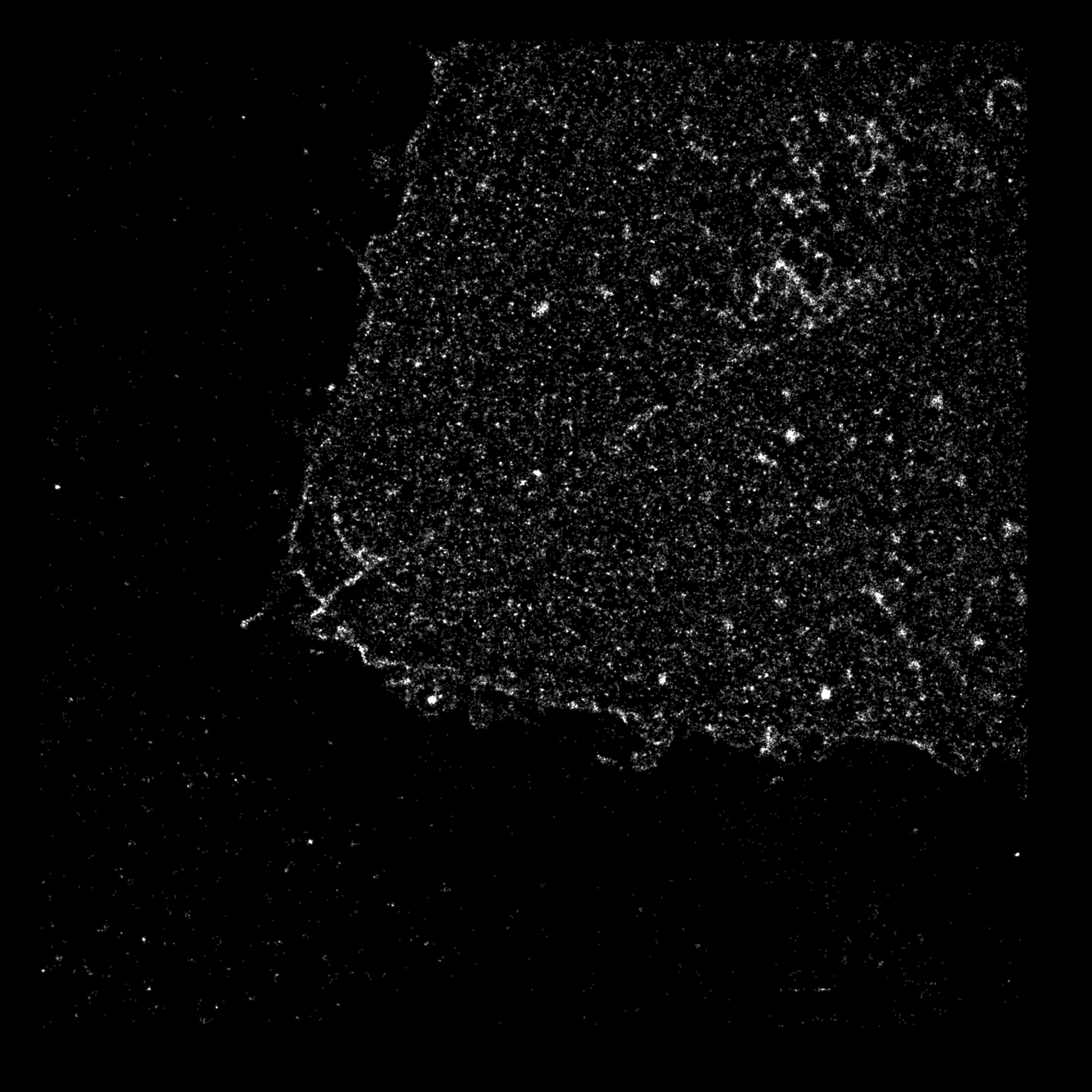

Supplement: Figure 2—source data 2. [file elife-101652-fig2-data2.zip › Figure 2-source data 2/Figure 2F-right.tif]

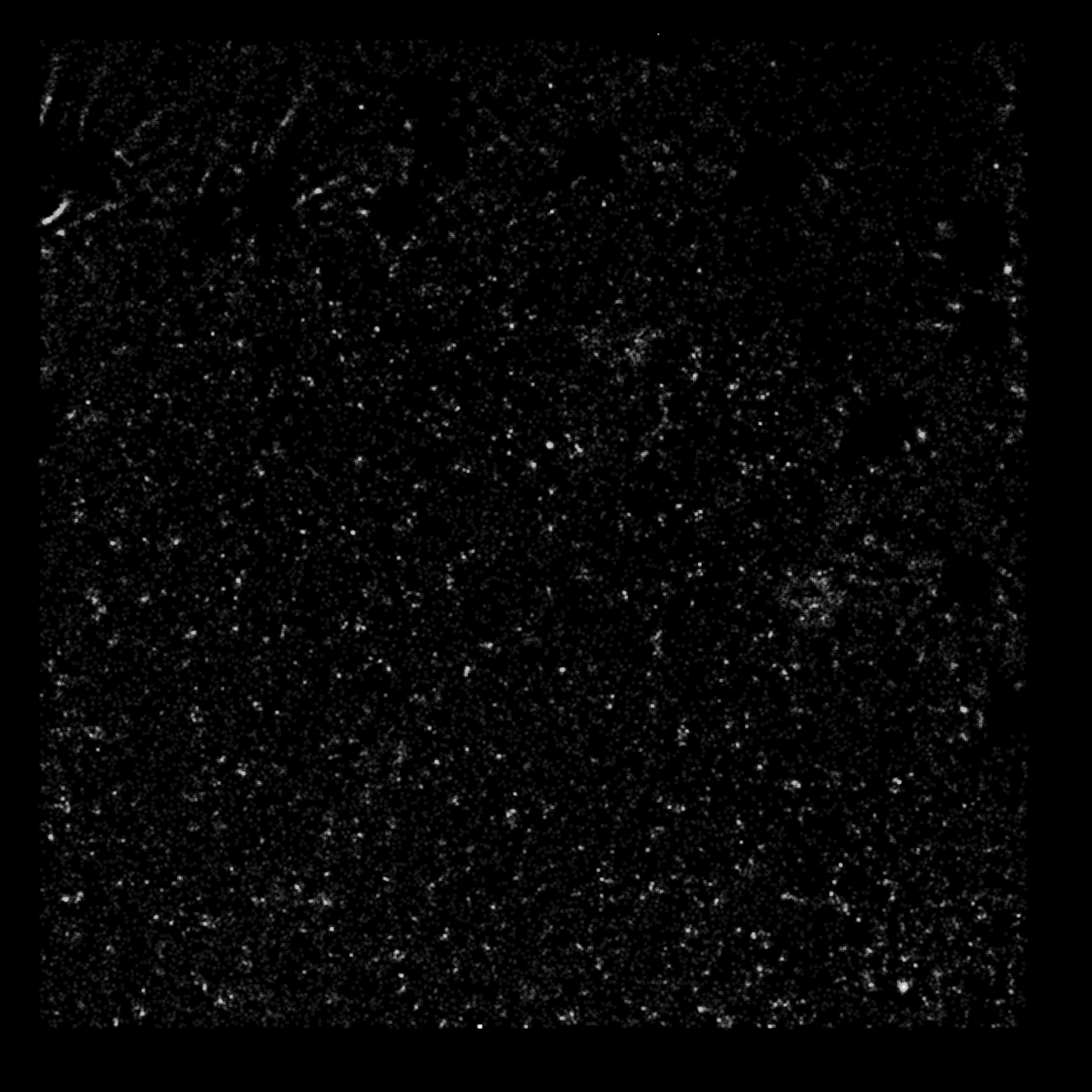

Supplement: Figure 2—source data 2. [file elife-101652-fig2-data2.zip › Figure 2-source data 2/Figure 2D.tif]

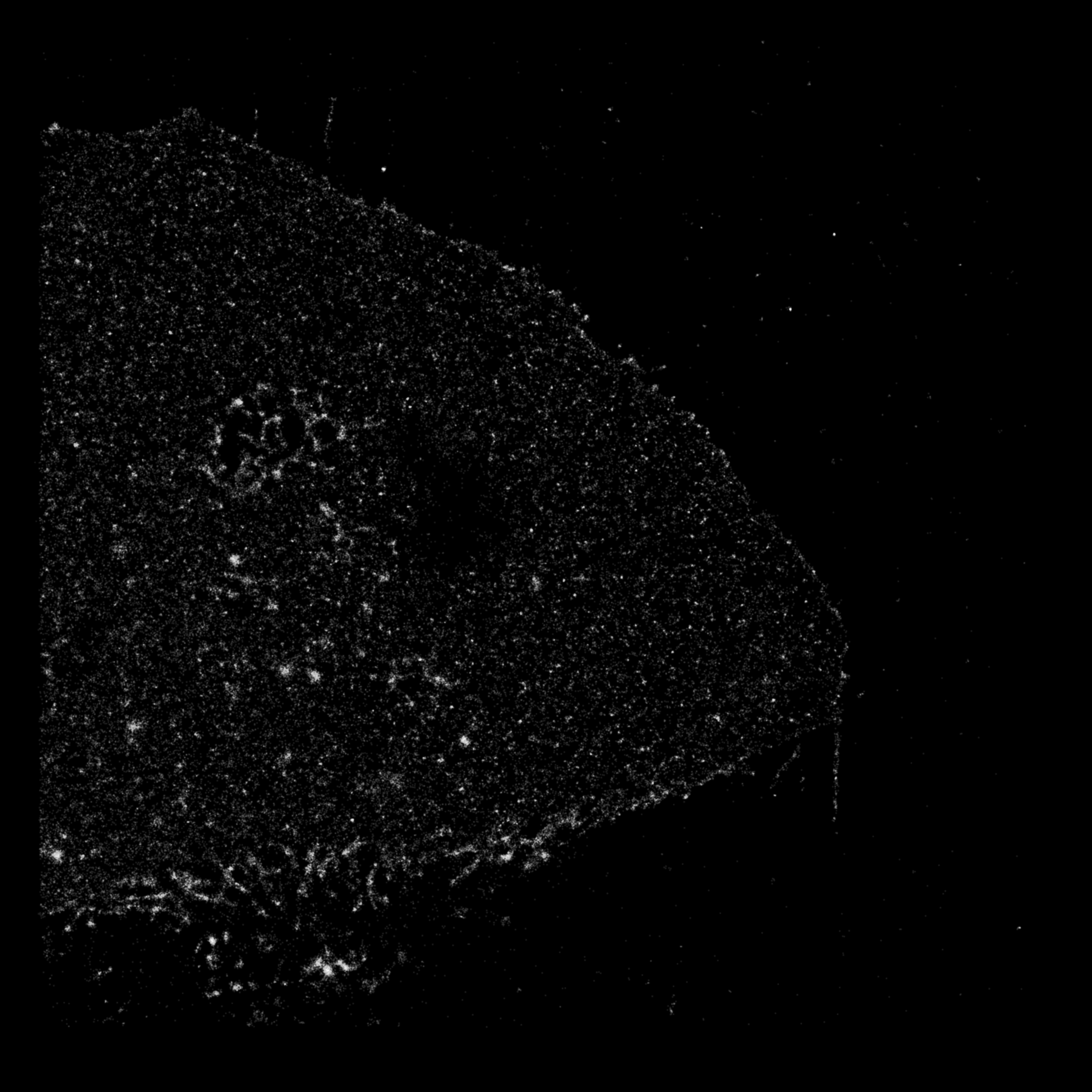

Supplement: Figure 2—source data 2. [file elife-101652-fig2-data2.zip › Figure 2-source data 2/Figure 2F-left-EGFR.tif]

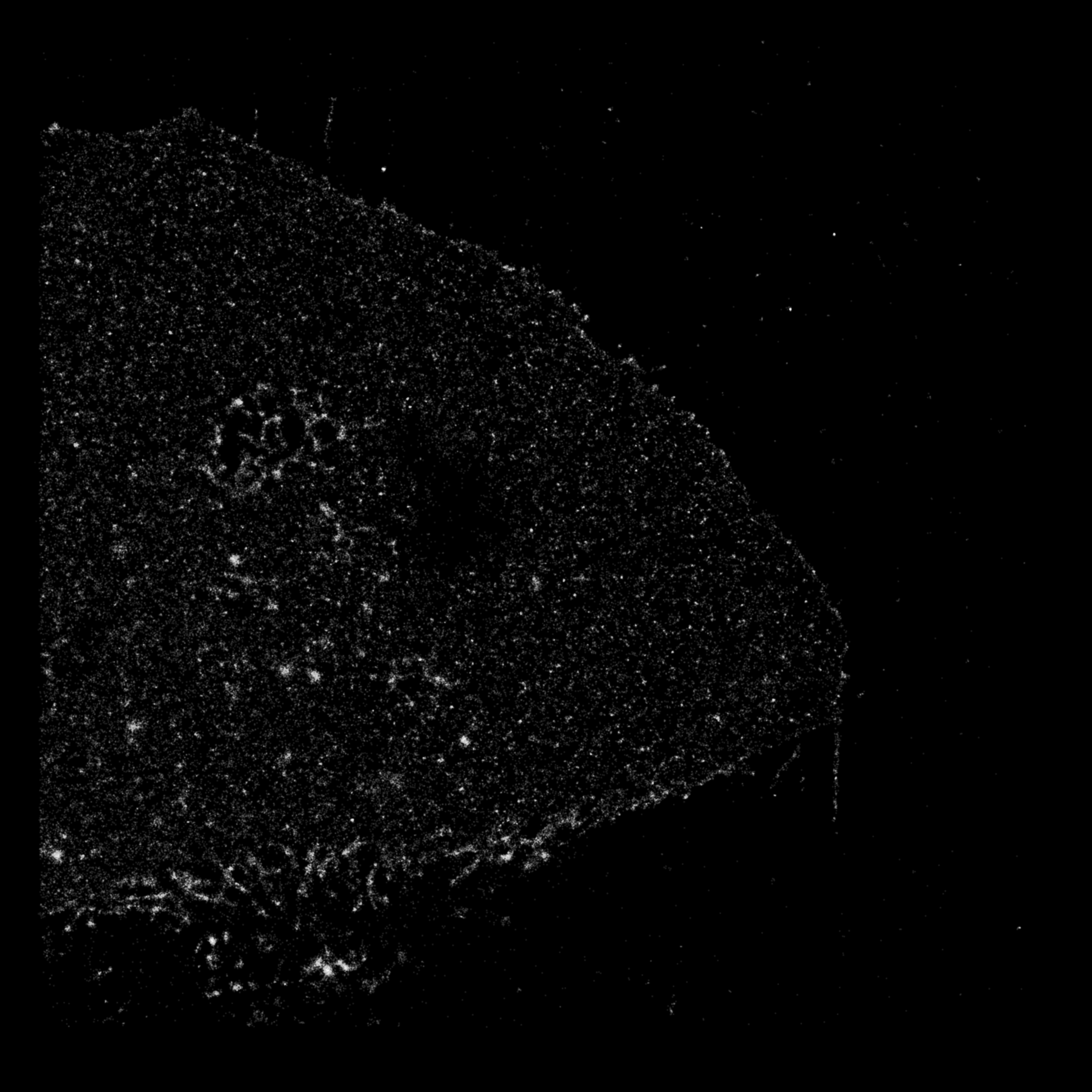

Supplement: Figure 2—source data 2. [file elife-101652-fig2-data2.zip › Figure 2-source data 2/Figure 2F-left.tif]

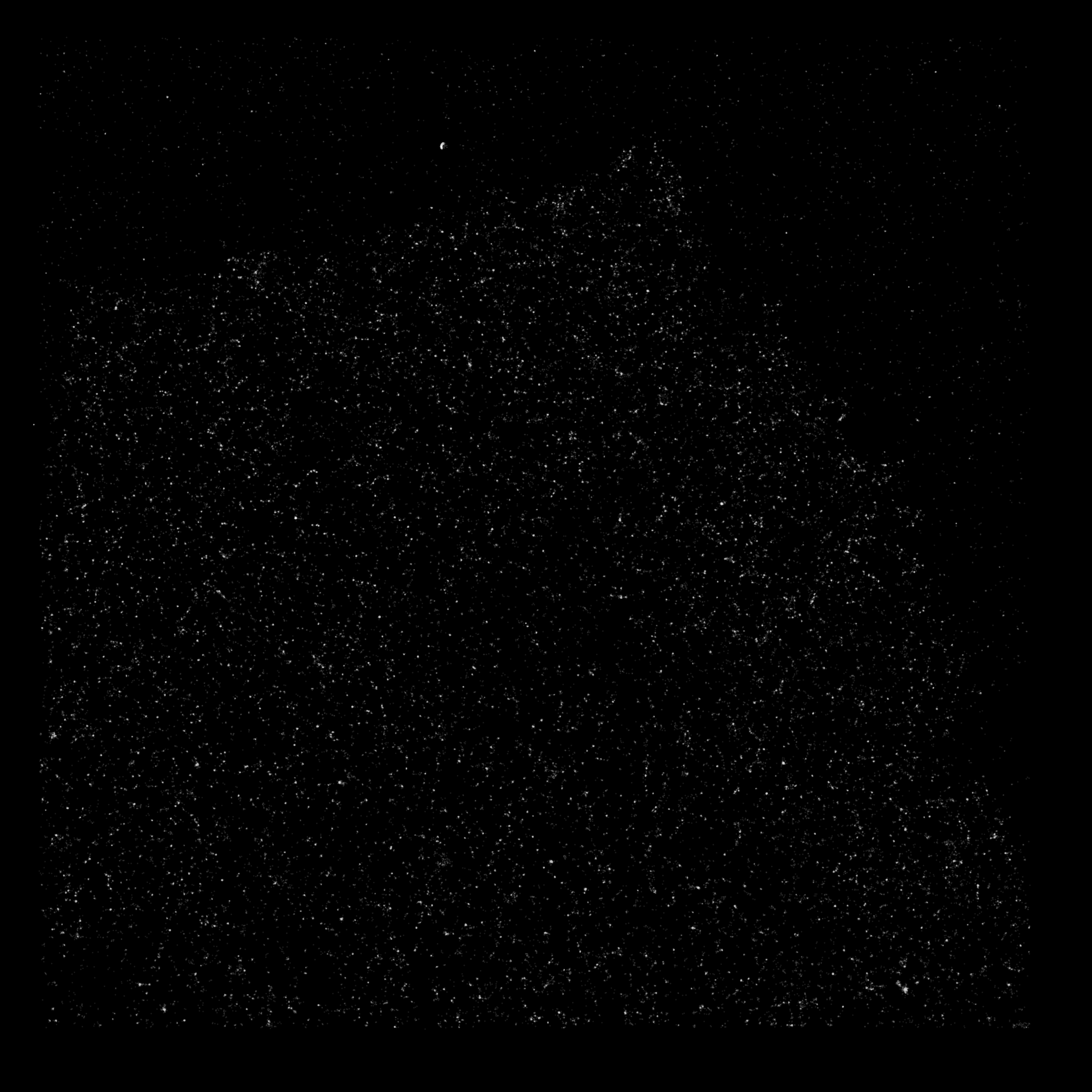

Supplement: Figure 2—source data 2. [file elife-101652-fig2-data2.zip › Figure 2-source data 2/Figure 2D-GRB2.tif]

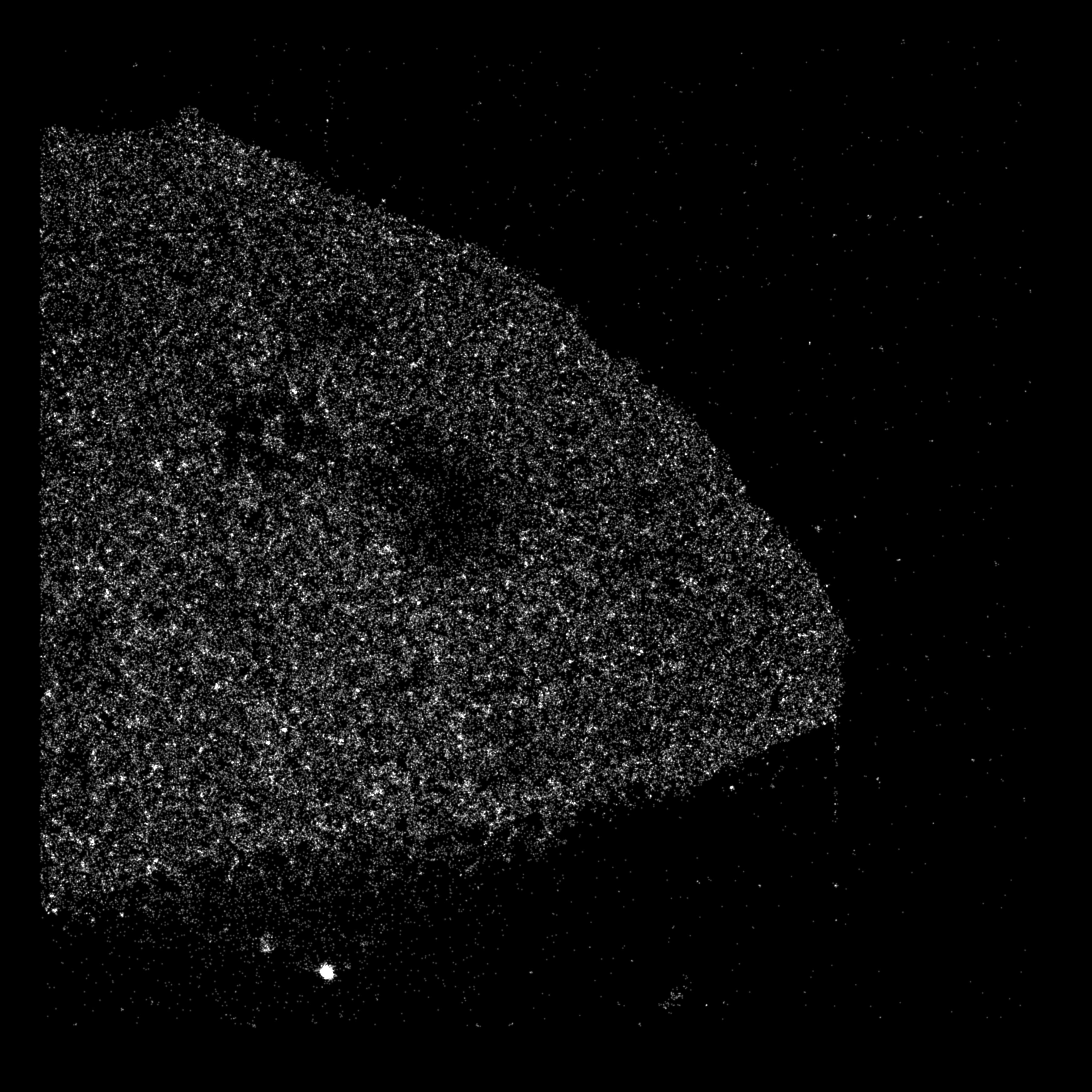

Supplement: Figure 2—source data 2. [file elife-101652-fig2-data2.zip › Figure 2-source data 2/Figure 2F-left-PI(3,4,5)P3.tif]

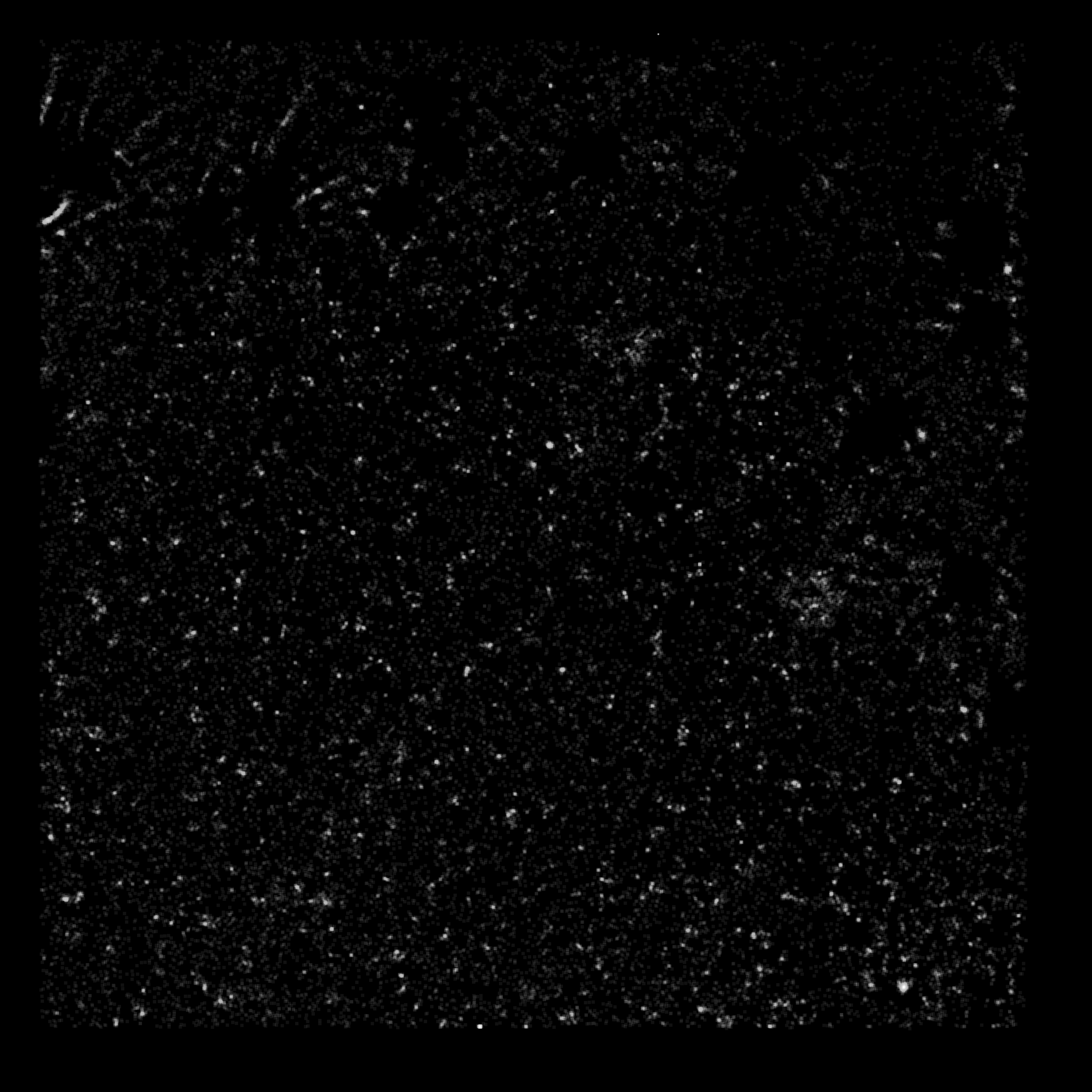

Supplement: Figure 2—source data 2. [file elife-101652-fig2-data2.zip › Figure 2-source data 2/Figure 2D-EGFR.tif]

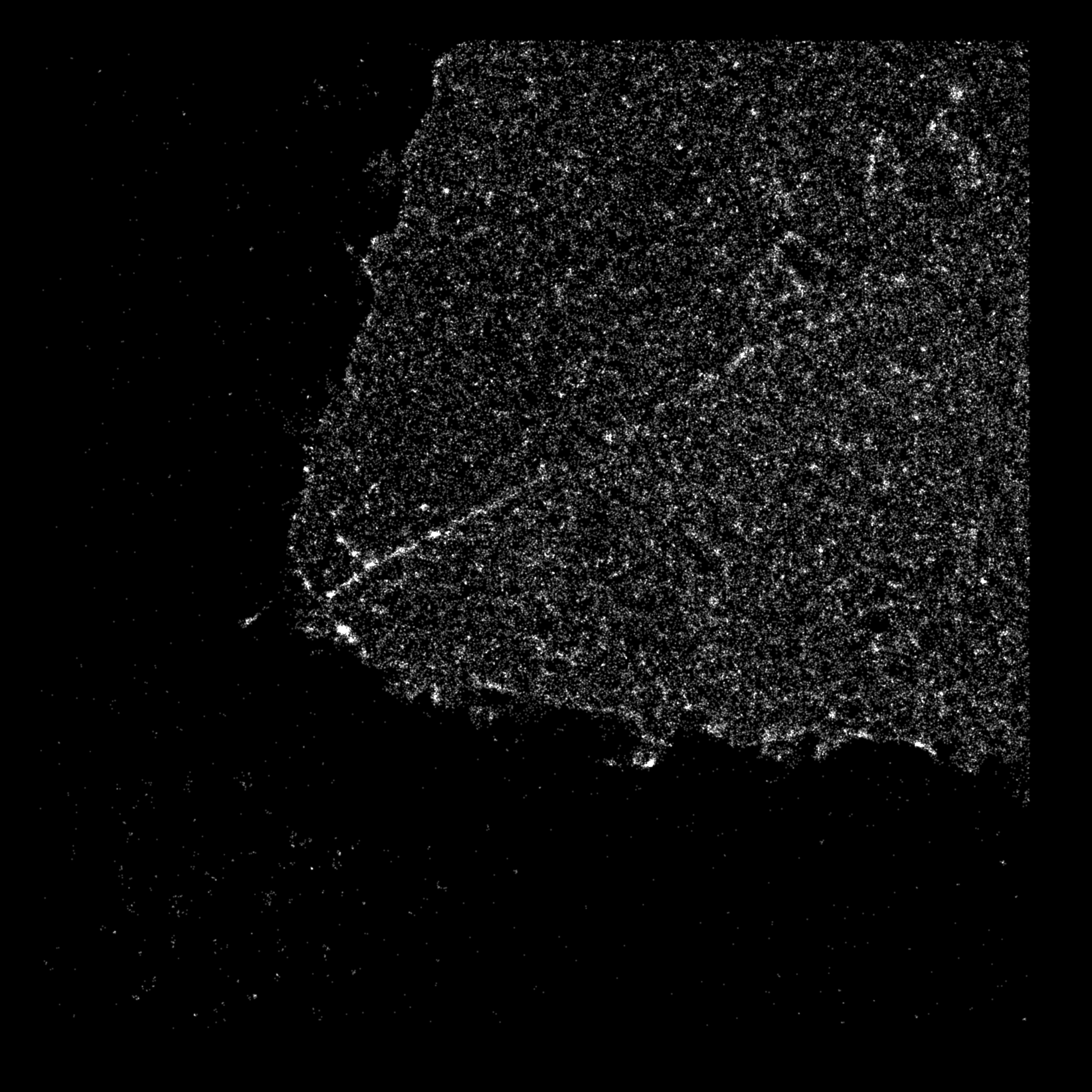

Supplement: Figure 2—source data 2. [file elife-101652-fig2-data2.zip › Figure 2-source data 2/Figure 2F-right-PI(3,4,5)P3.tif]

A

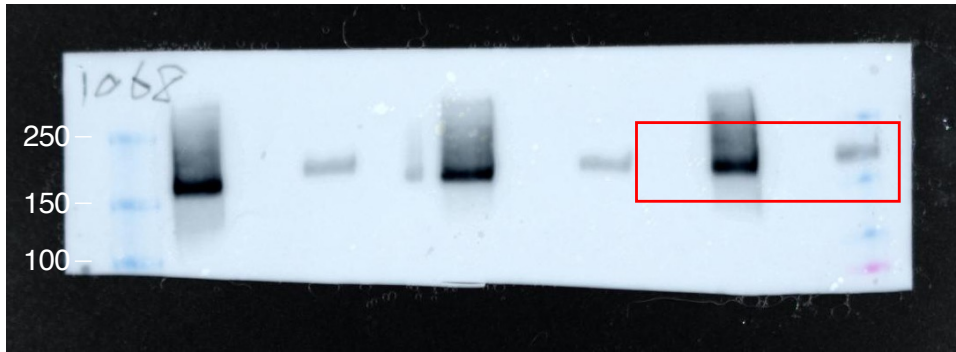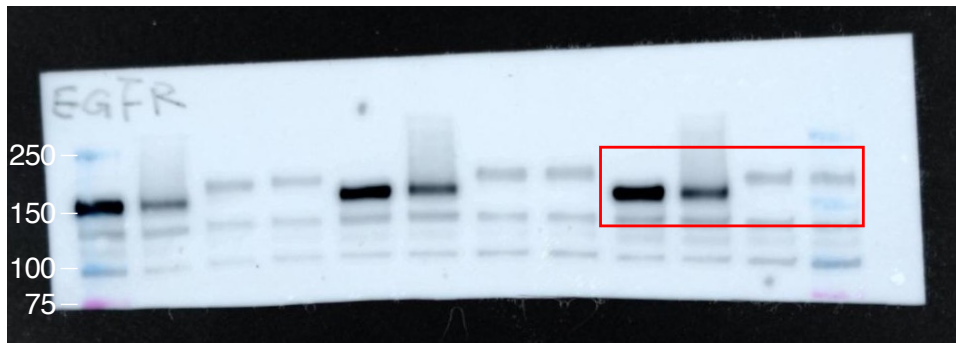

Supplement: Figure 3—figure supplement 1—source data 1. [file elife-101652-fig3-figsupp1-data1.zip › Figure 3-figure supplement 1-source data 1.pdf]

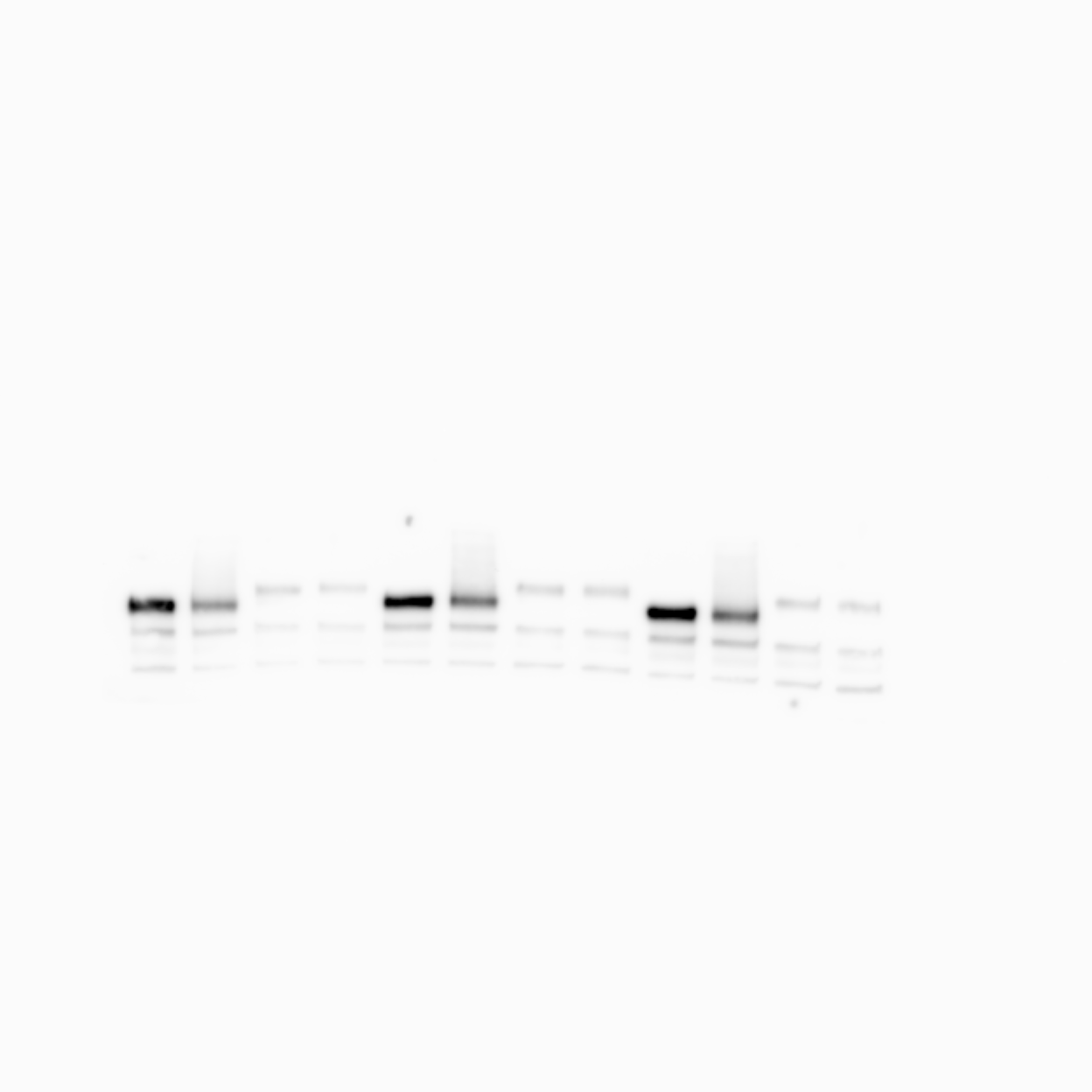

Supplement: Figure 3—figure supplement 1—source data 2. [file elife-101652-fig3-figsupp1-data2.zip › Figure 3-figure supplement 1-source data 2/Figure 3-supplement 1A-EGFR.tif]

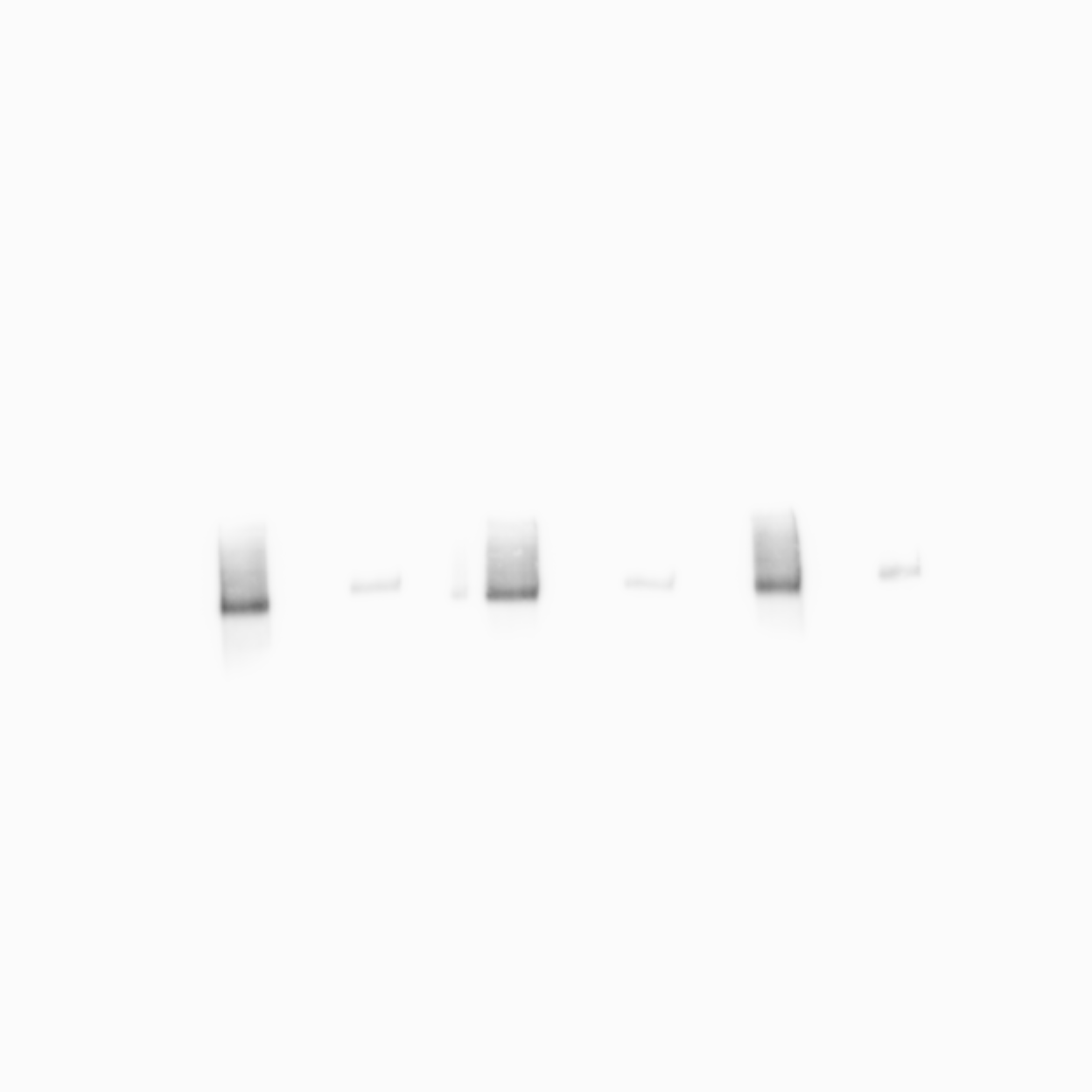

Supplement: Figure 3—figure supplement 1—source data 2. [file elife-101652-fig3-figsupp1-data2.zip › Figure 3-figure supplement 1-source data 2/Figure 3-supplement 1A-pY1068.tif]

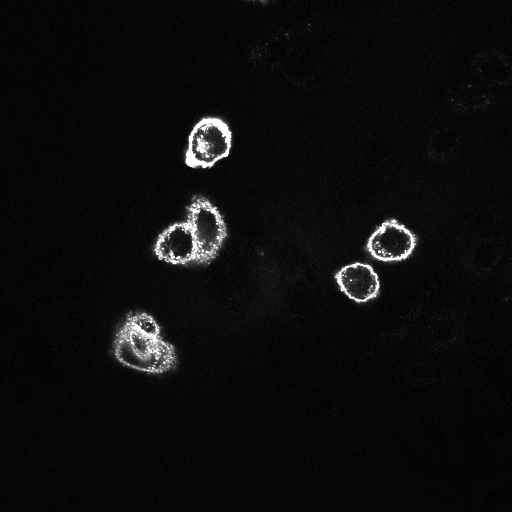

Supplement: Figure 4—figure supplement 2—source data 1. [file elife-101652-fig4-figsupp2-data1.zip › Figure 4-figure supplement 2-source data/Figure 4-supplement 4-3RN_Cy5EGF.tif]

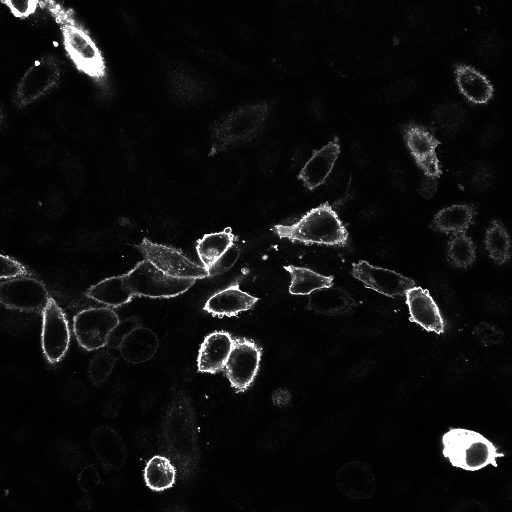

Supplement: Figure 4—figure supplement 2—source data 1. [file elife-101652-fig4-figsupp2-data1.zip › Figure 4-figure supplement 2-source data/Figure 4-supplement 2C-EGFRvIII_EGFR.tif]

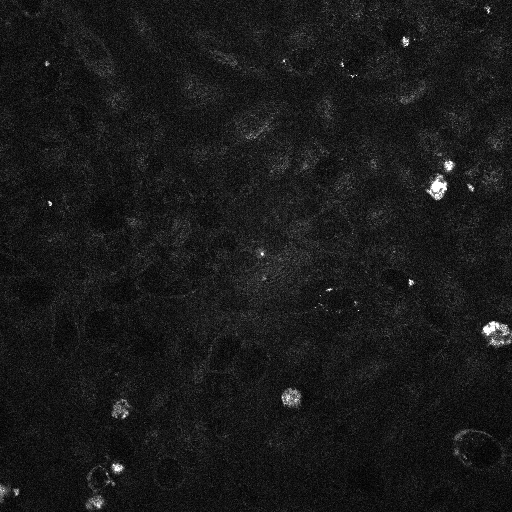

Supplement: Figure 4—figure supplement 2—source data 1. [file elife-101652-fig4-figsupp2-data1.zip › Figure 4-figure supplement 2-source data/Figure 4-supplement 2C-EGFRvIII_Cy5EGF.tif]

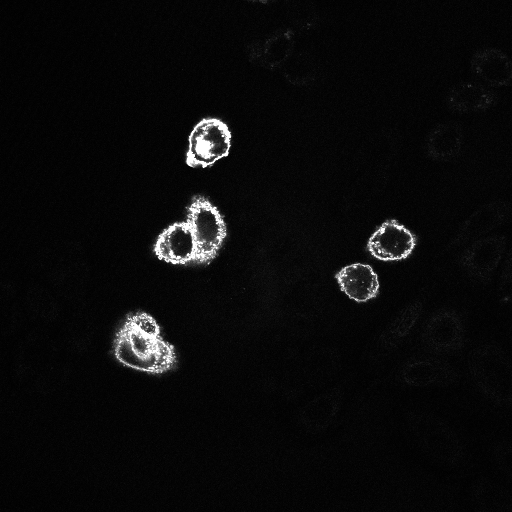

Supplement: Figure 4—figure supplement 2—source data 1. [file elife-101652-fig4-figsupp2-data1.zip › Figure 4-figure supplement 2-source data/Figure 4-supplement 2C-3RN_EGFR.tif]

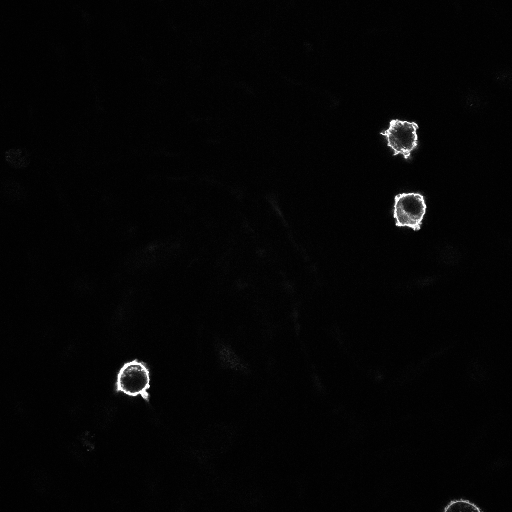

Supplement: Figure 4—figure supplement 2—source data 1. [file elife-101652-fig4-figsupp2-data1.zip › Figure 4-figure supplement 2-source data/Figure 4-supplement 2C-WT_EGFR.tif]

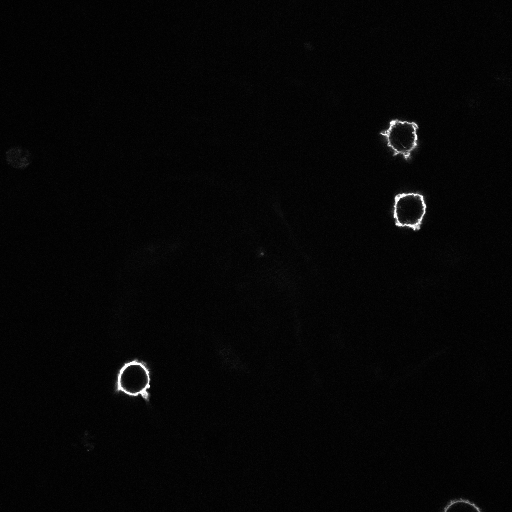

Supplement: Figure 4—figure supplement 2—source data 1. [file elife-101652-fig4-figsupp2-data1.zip › Figure 4-figure supplement 2-source data/Figure 4-supplement 2C-WT_Cy5EGF.tif]

A

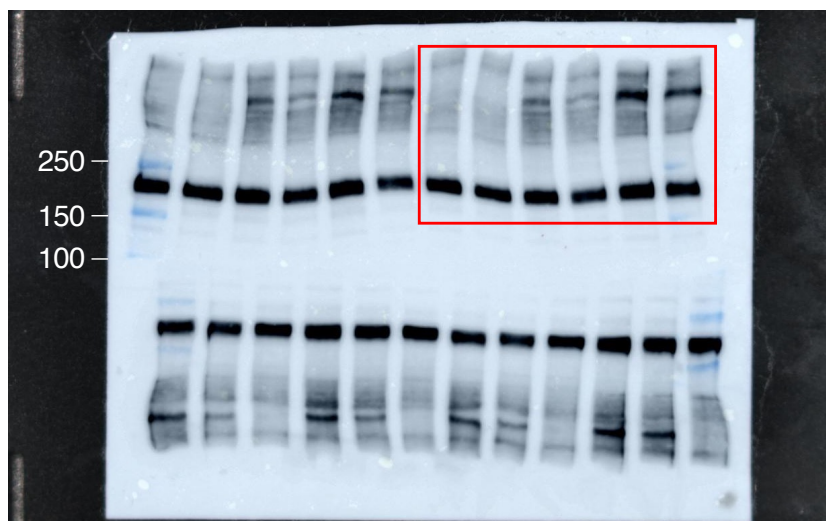

C

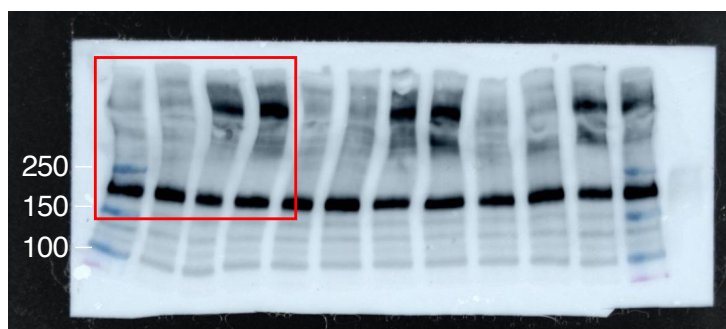

E

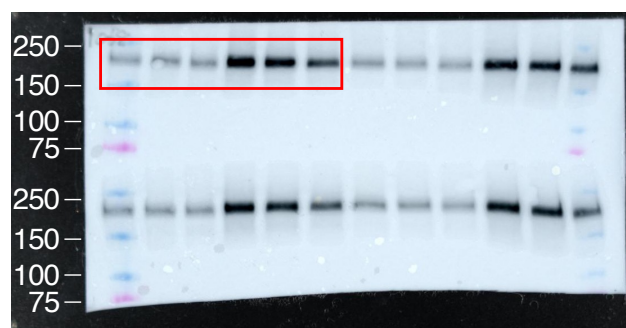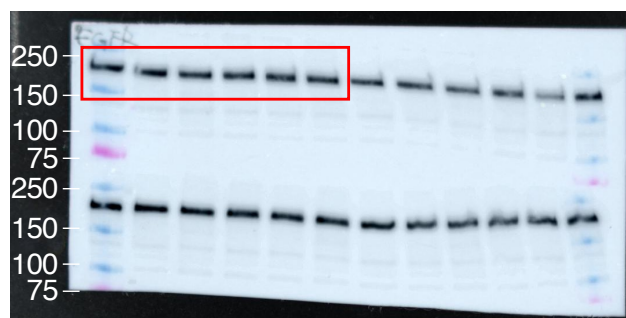

Supplement: Figure 5—source data 1. [file elife-101652-fig5-data1.zip › Figure 5-source data 1.pdf]

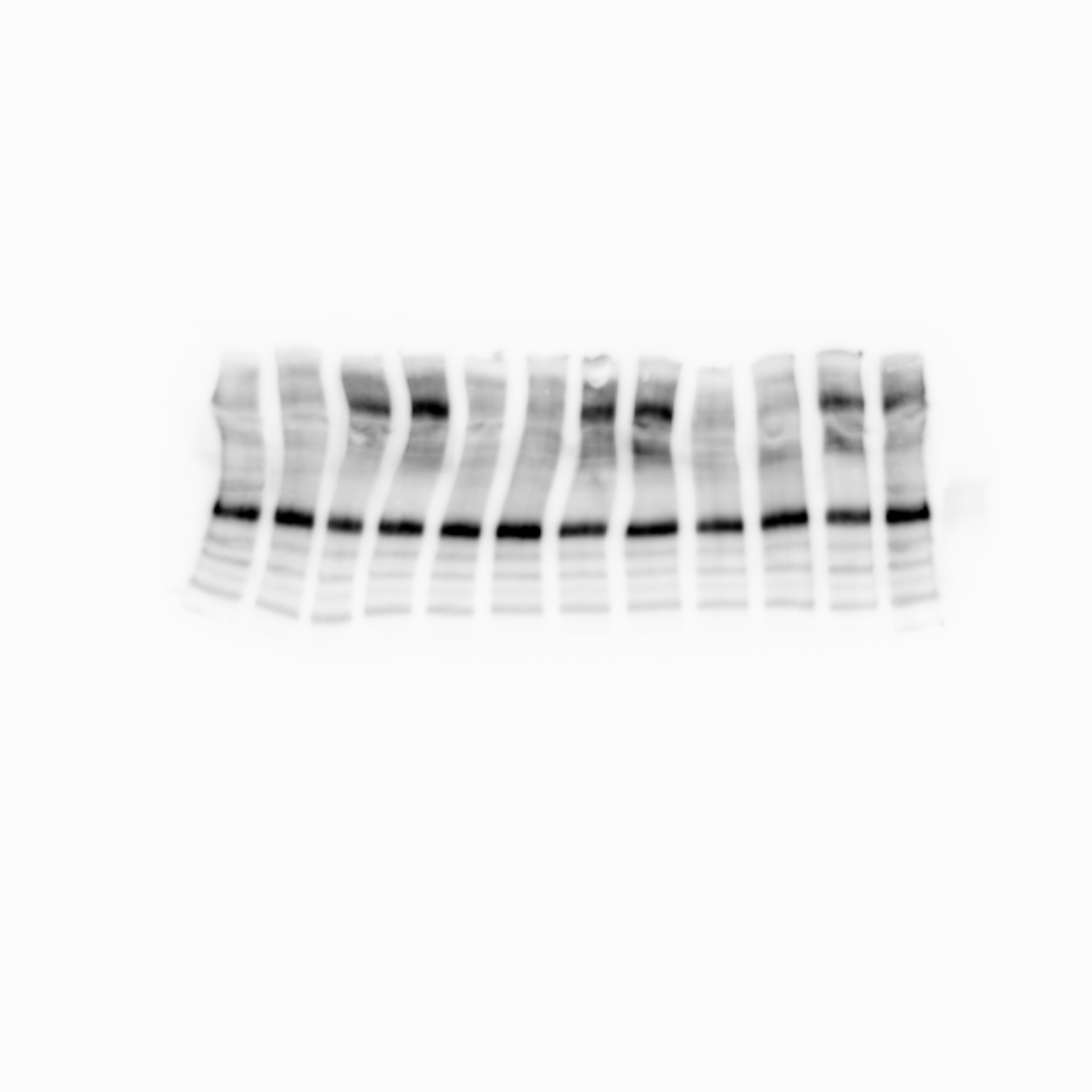

Supplement: Figure 5—source data 2. [file elife-101652-fig5-data2.zip › Figure 5-source data 2/Figure 5C.tif]

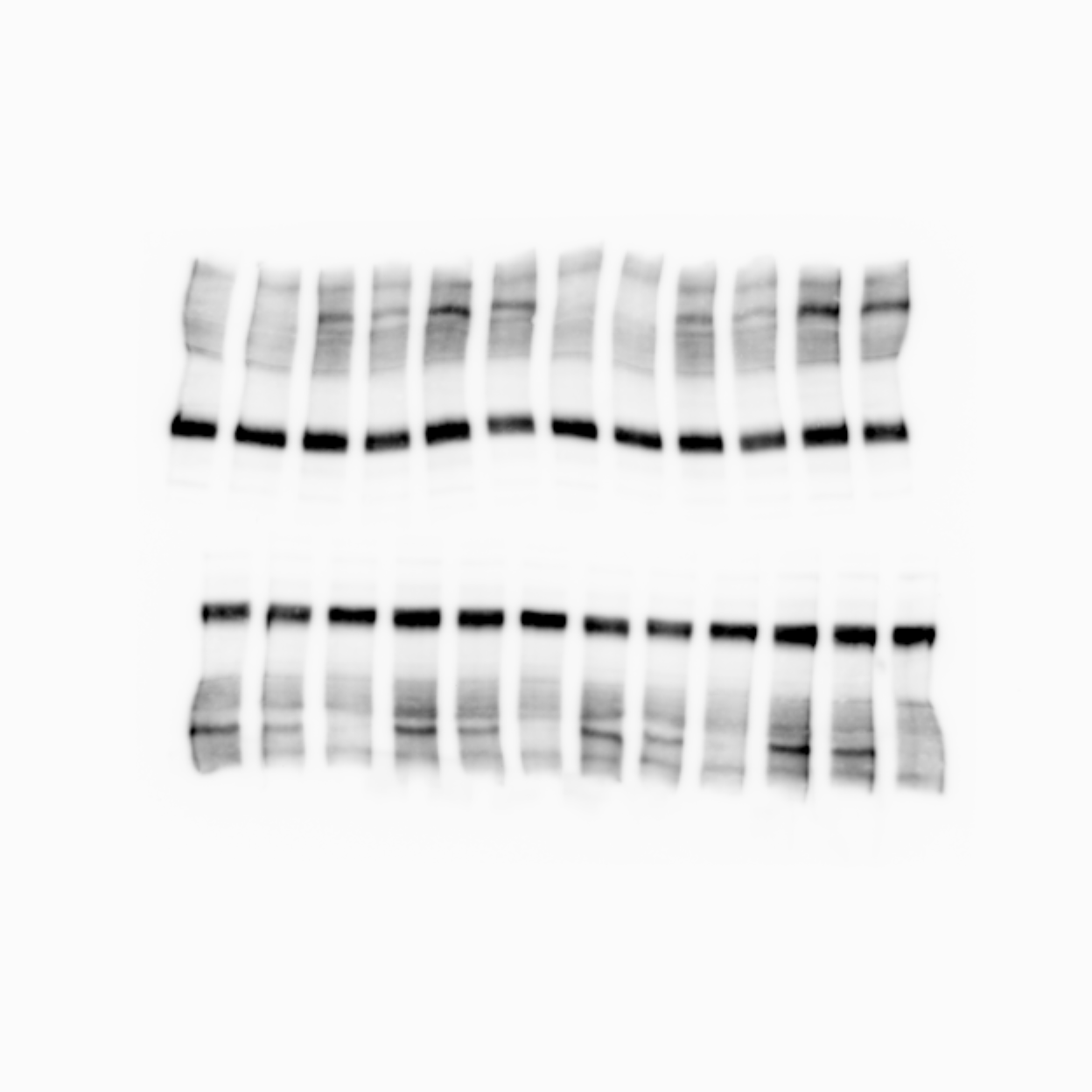

Supplement: Figure 5—source data 2. [file elife-101652-fig5-data2.zip › Figure 5-source data 2/Figure 5A.tif]

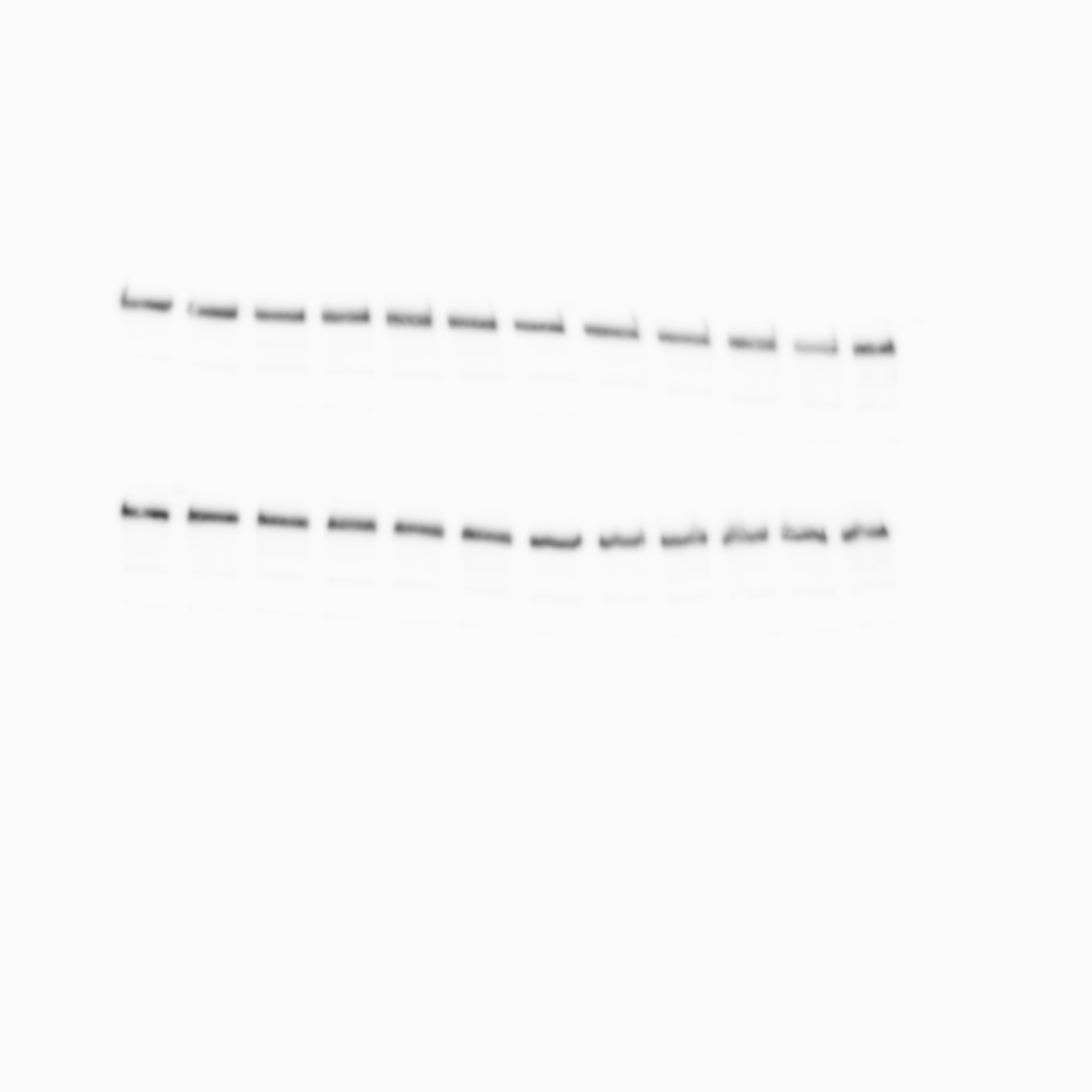

Supplement: Figure 5—source data 2. [file elife-101652-fig5-data2.zip › Figure 5-source data 2/Figure 5E-EGFR.tif]

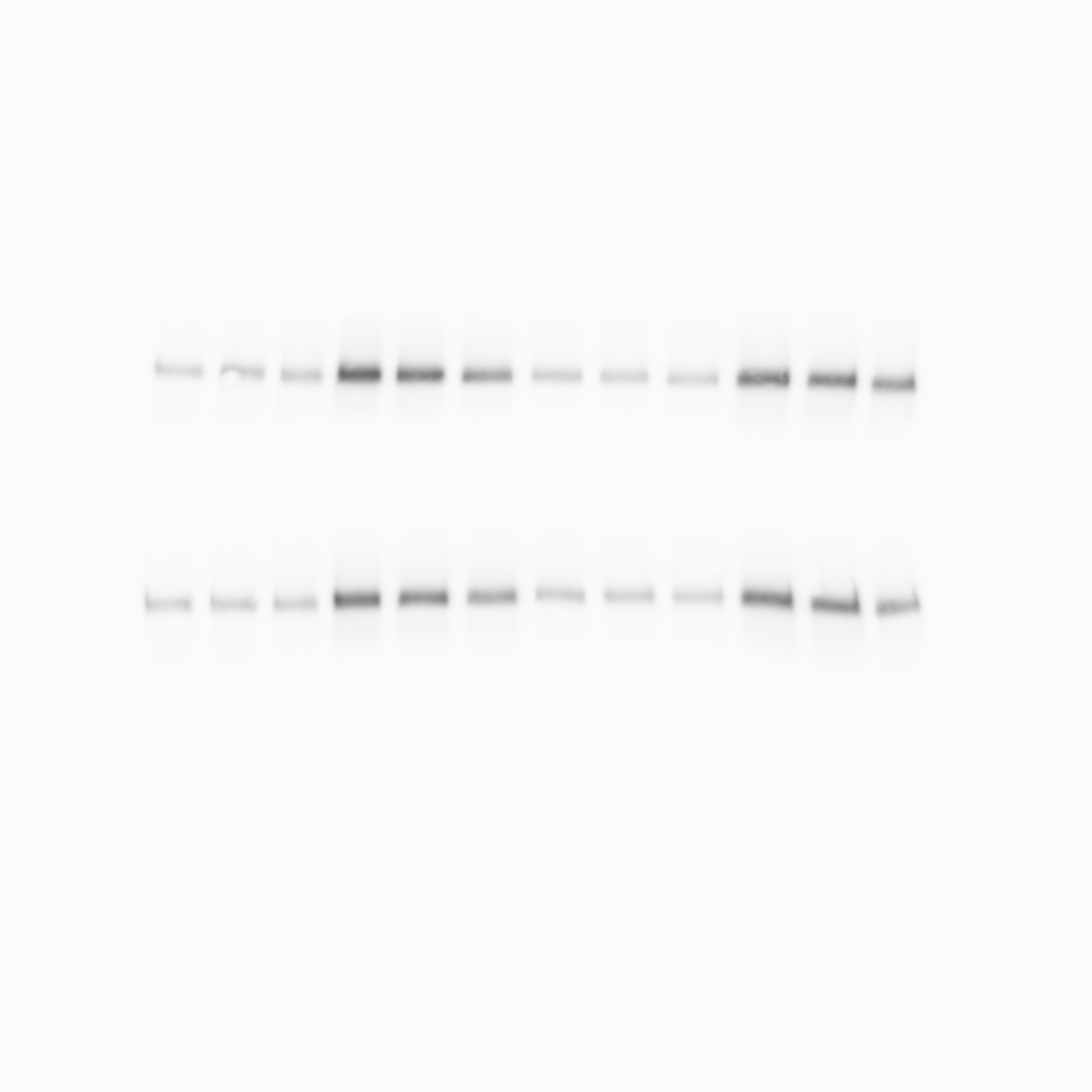

Supplement: Figure 5—source data 2. [file elife-101652-fig5-data2.zip › Figure 5-source data 2/Figure 5E-pY1068.tif]

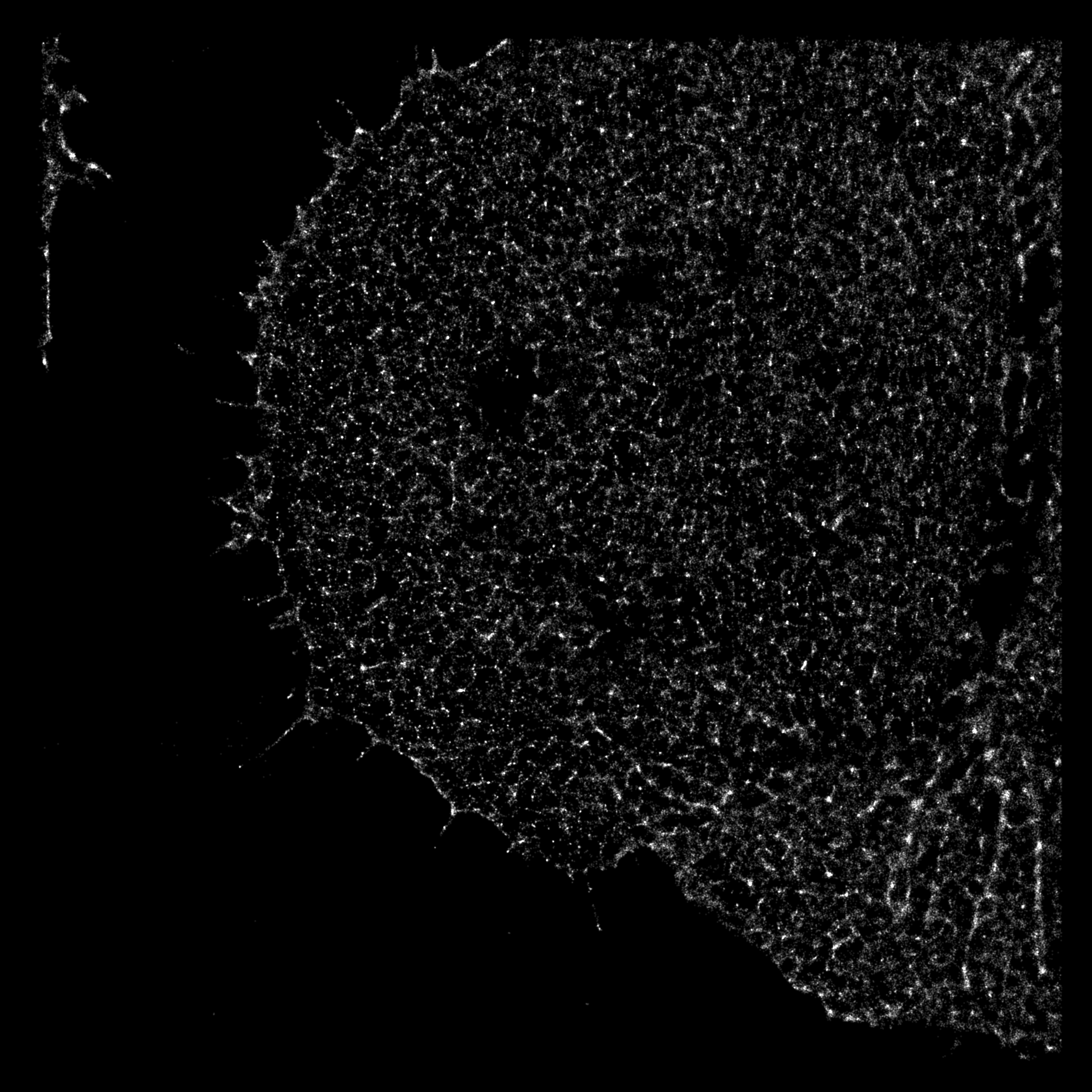

Supplement: Figure 6—source data 2. [file elife-101652-fig6-data2.zip › Figure 6-source data 2/Figure 6E-DN PLCG.tif]

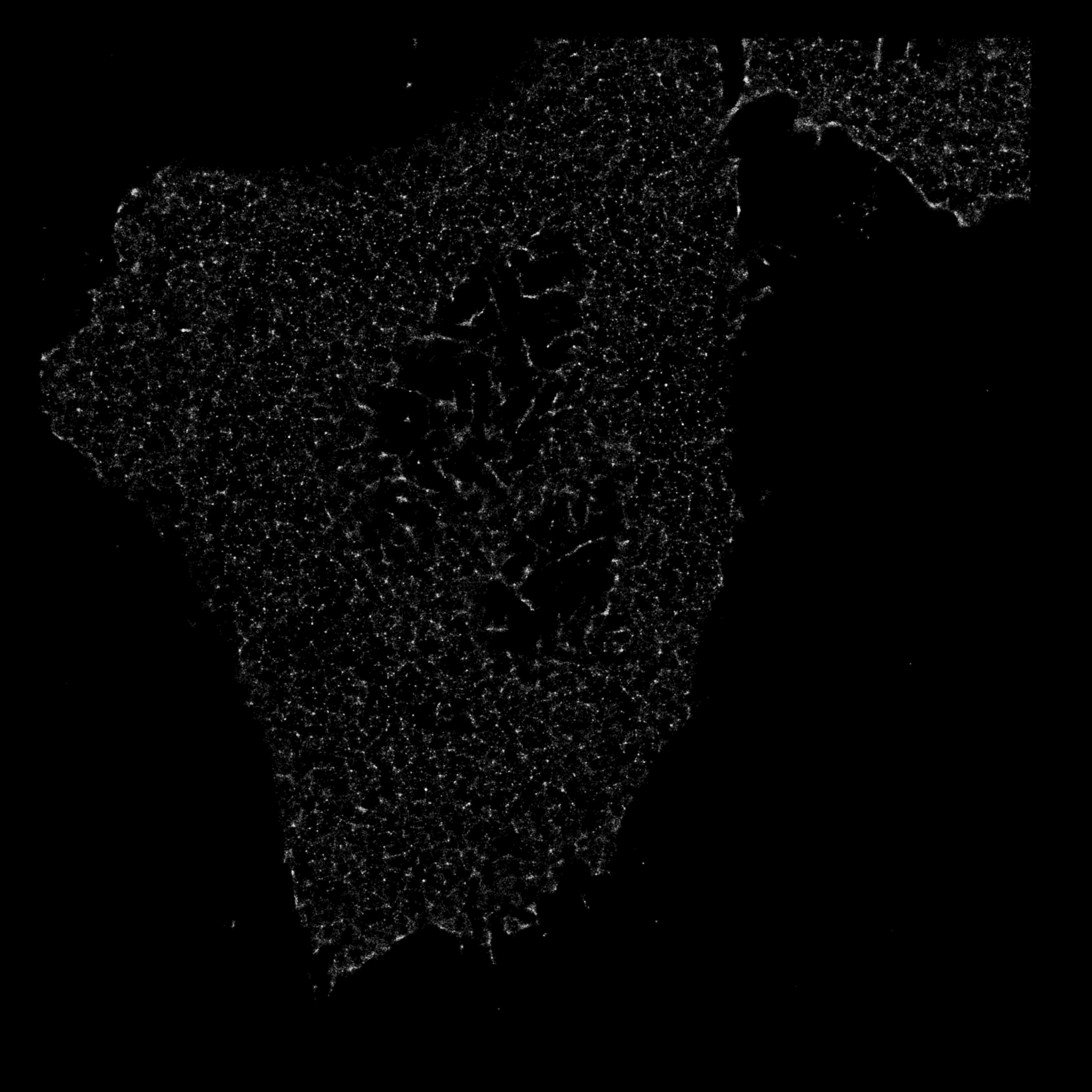

Supplement: Figure 6—source data 2. [file elife-101652-fig6-data2.zip › Figure 6-source data 2/Figure 6E-control-pY1068.tif]

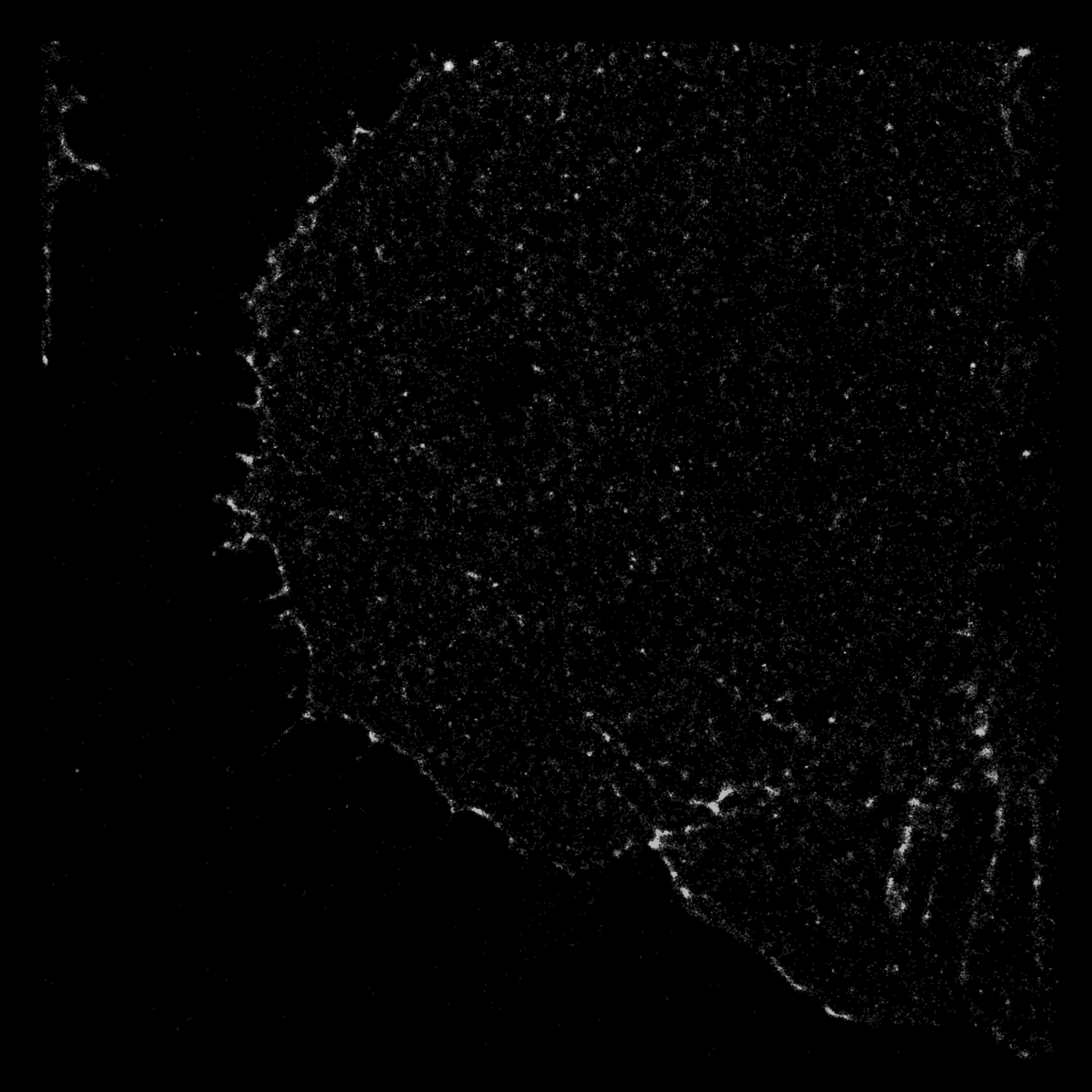

Supplement: Figure 6—source data 2. [file elife-101652-fig6-data2.zip › Figure 6-source data 2/Figure 6E-DN PLCG-PI(4,5)P2.tif]

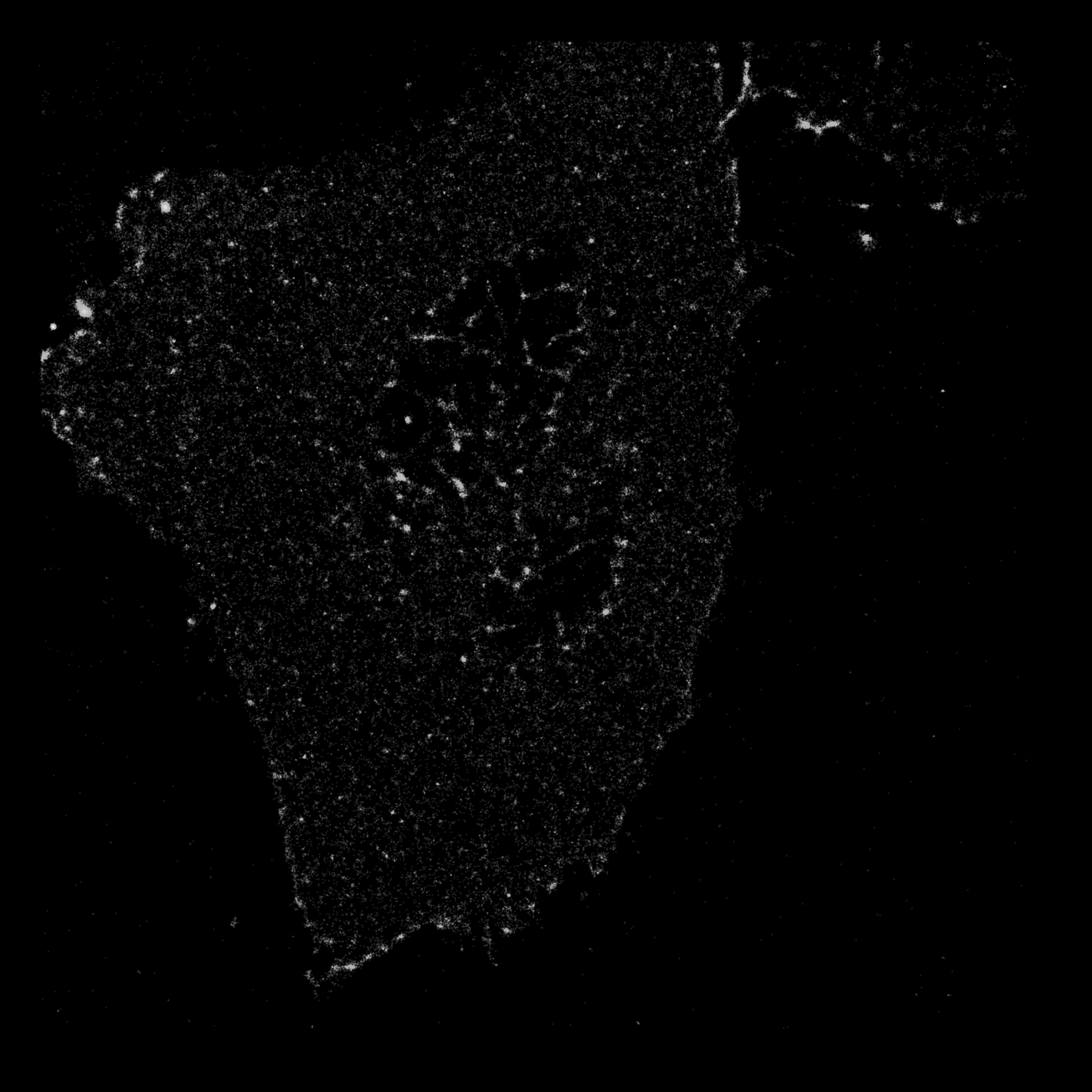

Supplement: Figure 6—source data 2. [file elife-101652-fig6-data2.zip › Figure 6-source data 2/Figure 6E-control-PI(4,5)P2.tif]

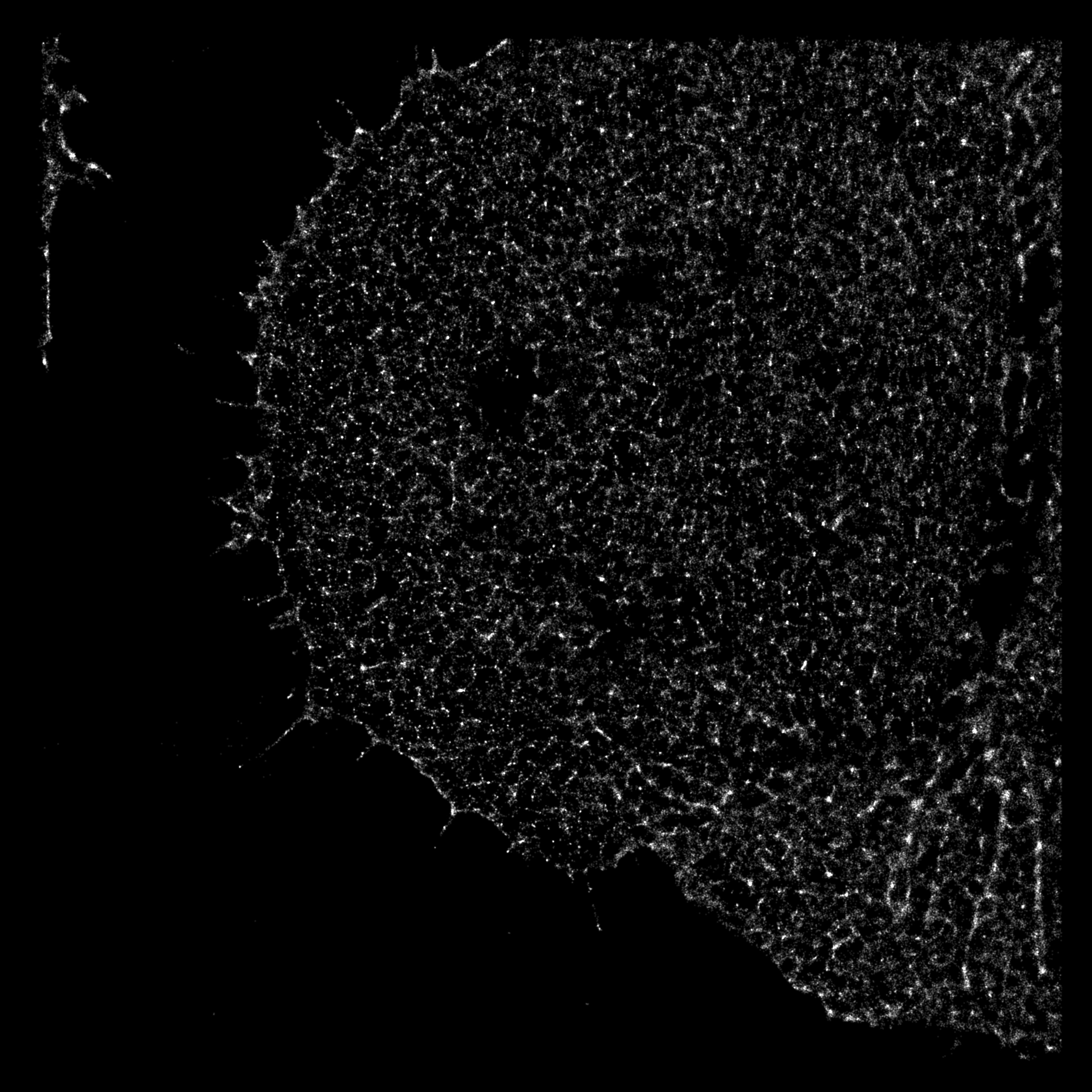

Supplement: Figure 6—source data 2. [file elife-101652-fig6-data2.zip › Figure 6-source data 2/Figure 6E-DN PLCG-pY1068.tif]

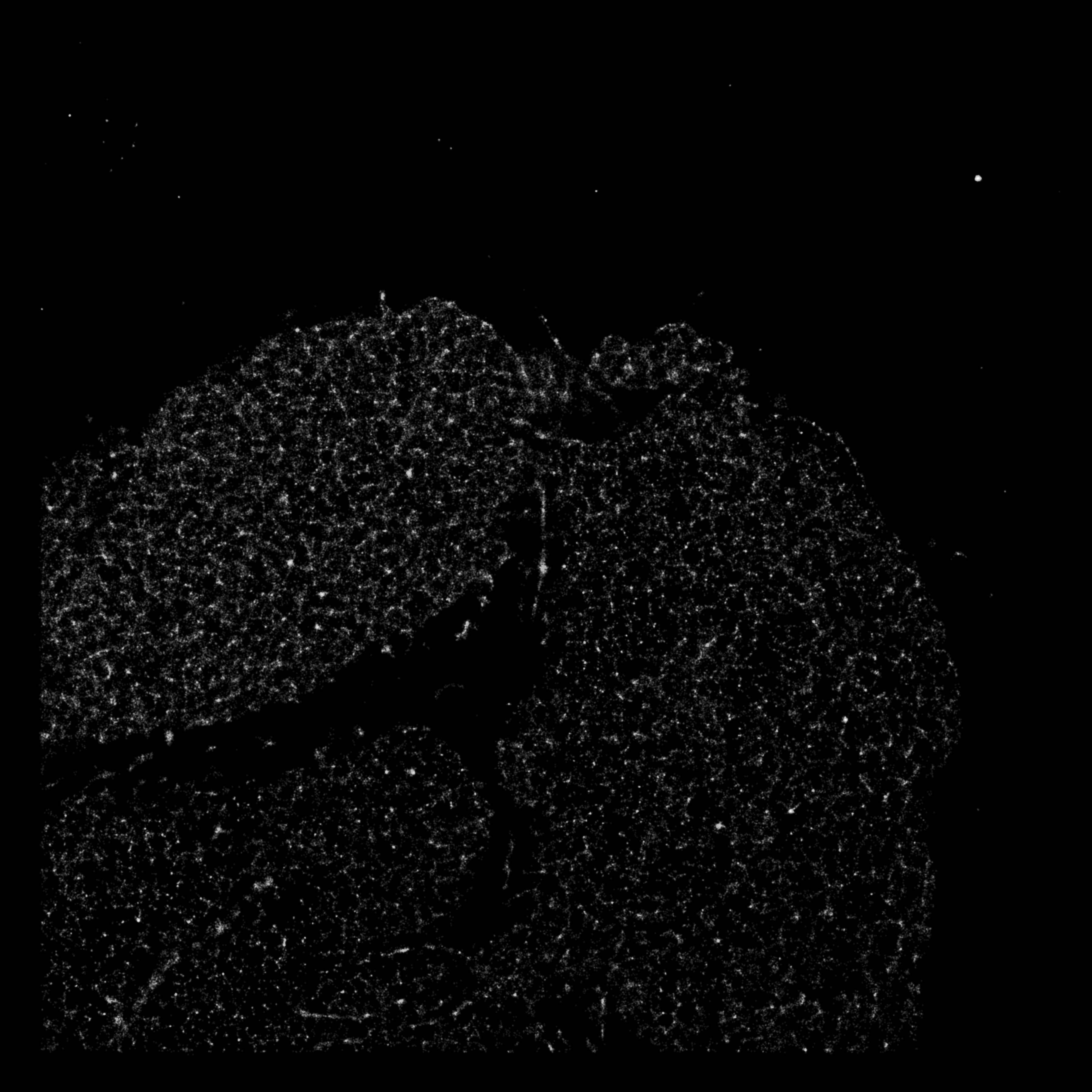

Supplement: Figure 6—source data 2. [file elife-101652-fig6-data2.zip › Figure 6-source data 2/Figure 6E-wort-pY1068.tif]

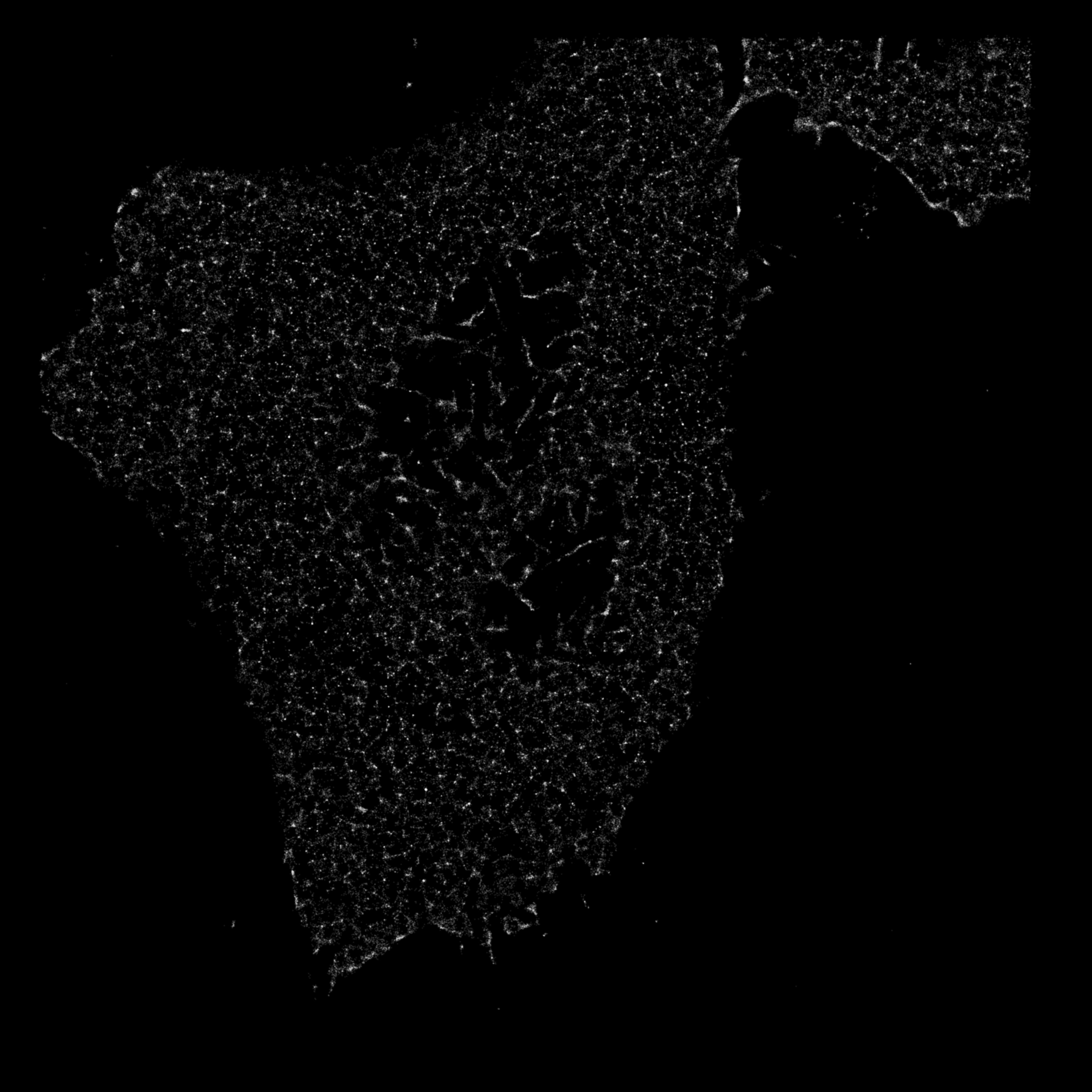

Supplement: Figure 6—source data 2. [file elife-101652-fig6-data2.zip › Figure 6-source data 2/Figure 6E-control.tif]

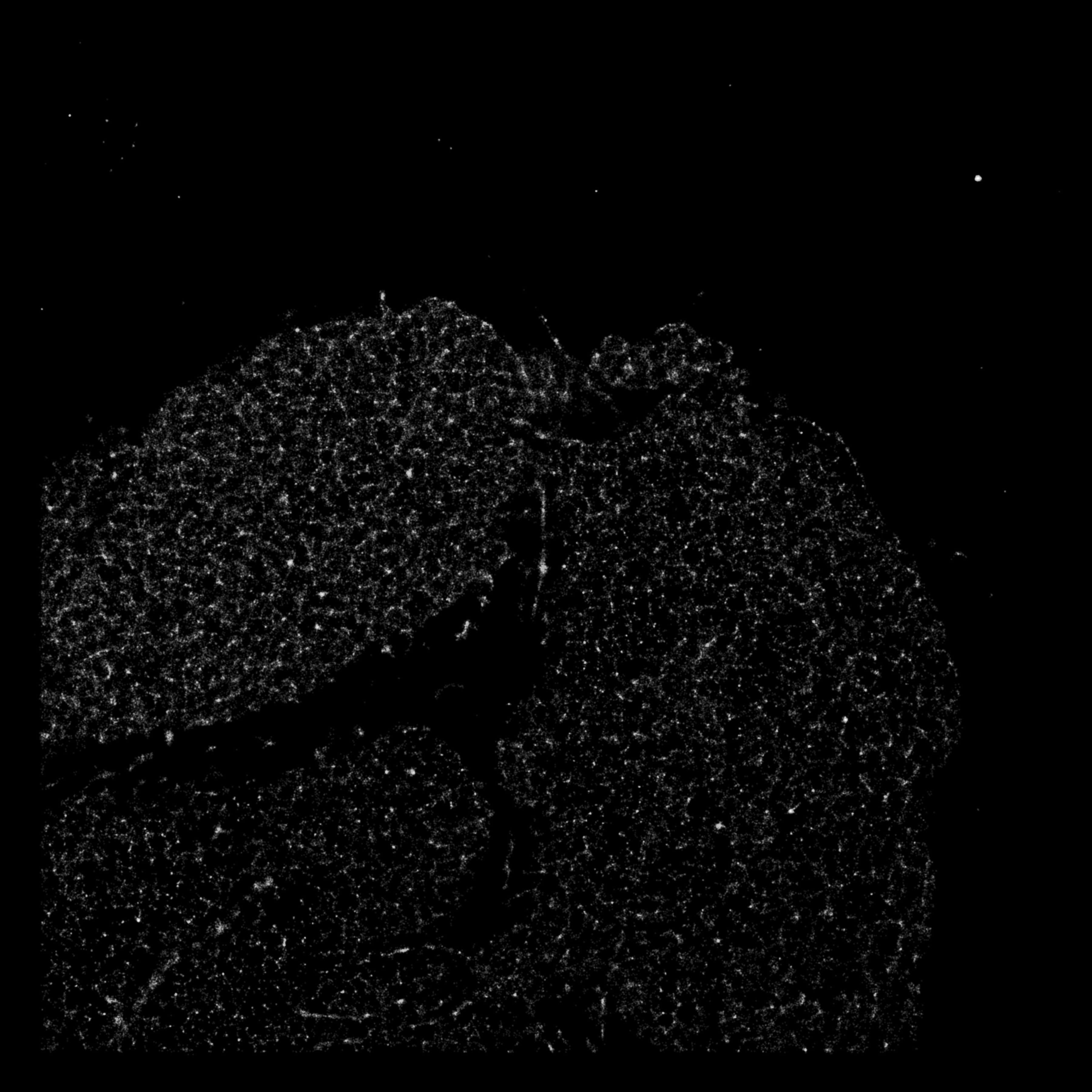

Supplement: Figure 6—source data 2. [file elife-101652-fig6-data2.zip › Figure 6-source data 2/Figure 6E-wort.tif]

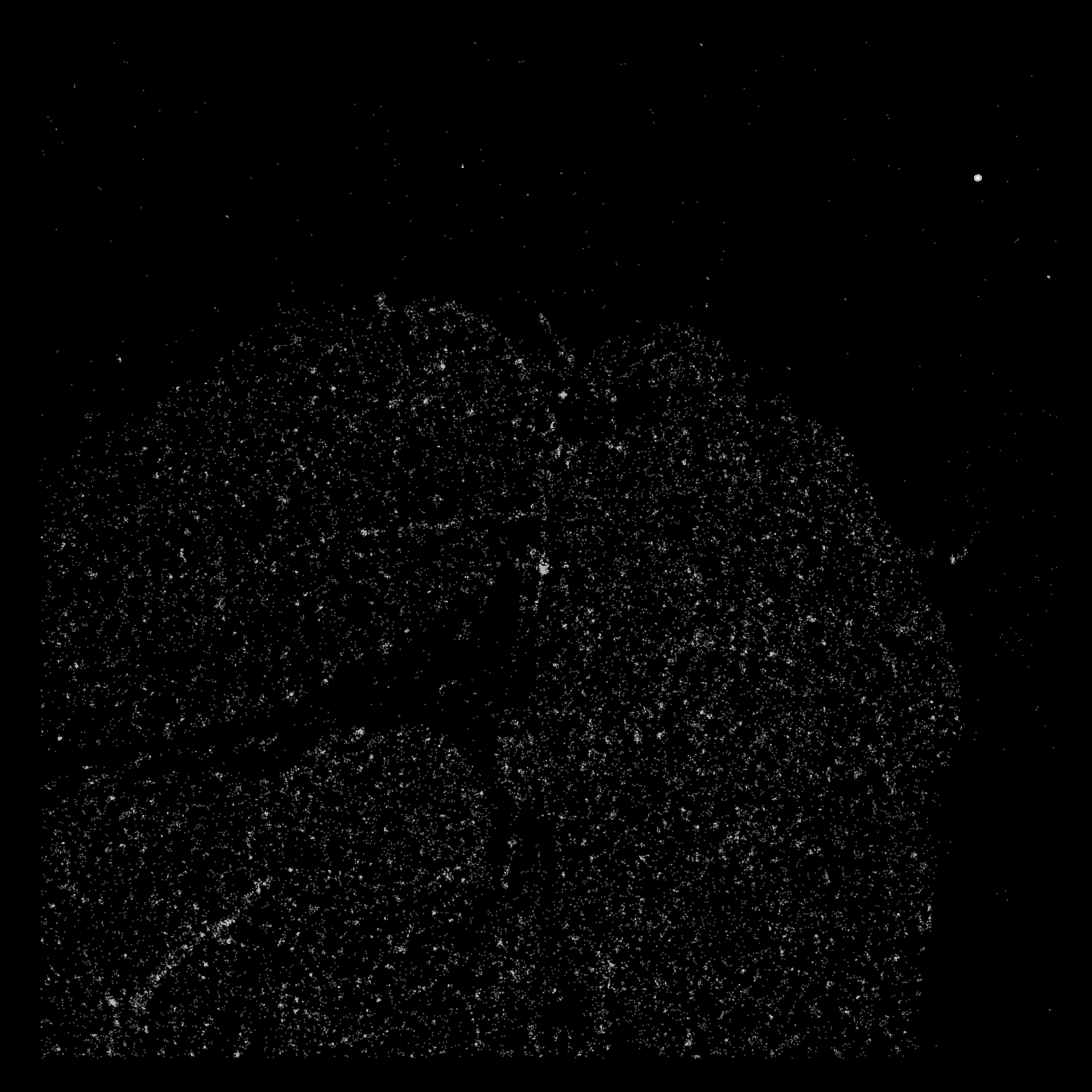

Supplement: Figure 6—source data 2. [file elife-101652-fig6-data2.zip › Figure 6-source data 2/Figure 6E-wort-PI(4,5)P2.tif]

C

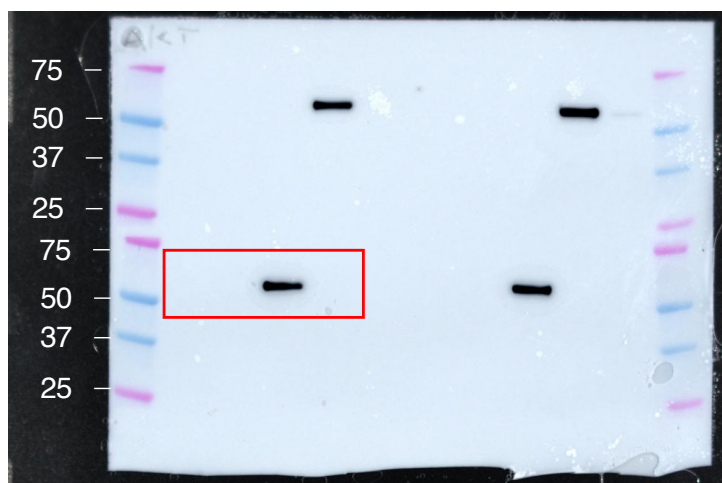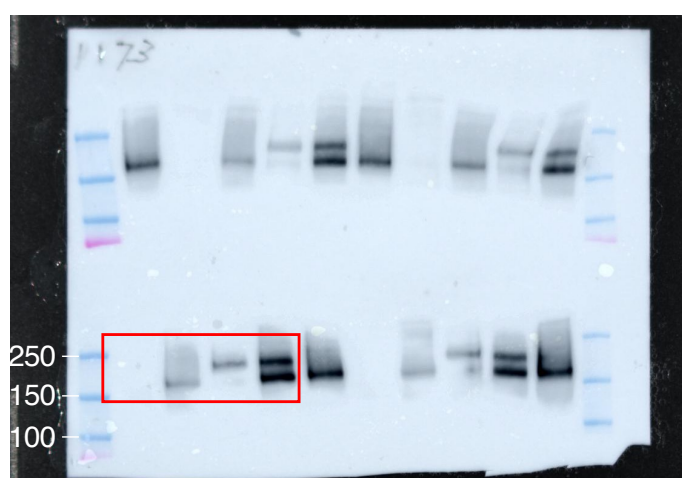

Supplement: Figure 6—figure supplement 1—source data 2. [file elife-101652-fig6-figsupp1-data2.zip › Figure 6-figure supplement 1-source data 2.pdf]

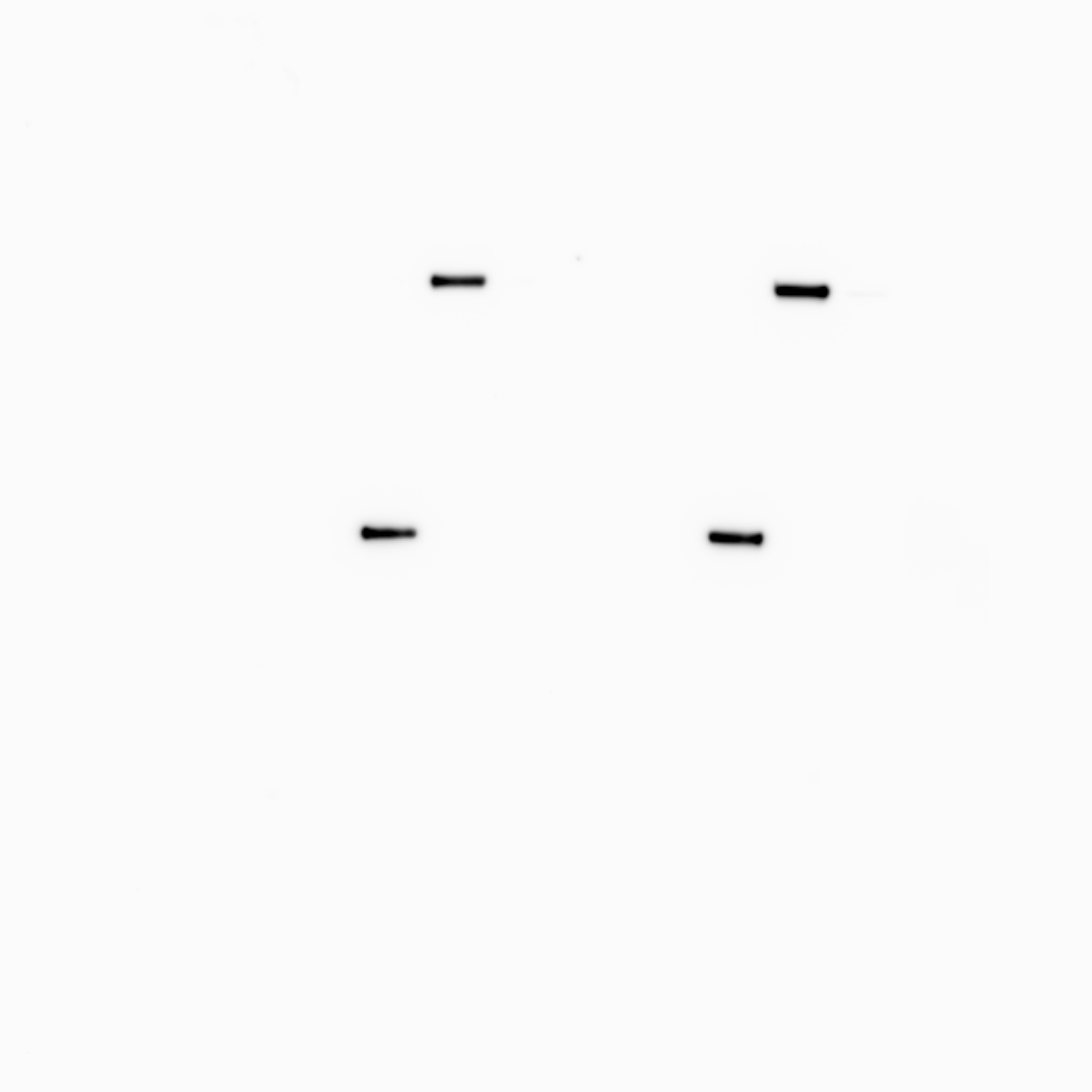

Supplement: Figure 6—figure supplement 1—source data 3. [file elife-101652-fig6-figsupp1-data3.zip › Figure 6-figure supplement 1-source data 3/Figure 6-supplement 1C-pAKT.tif]

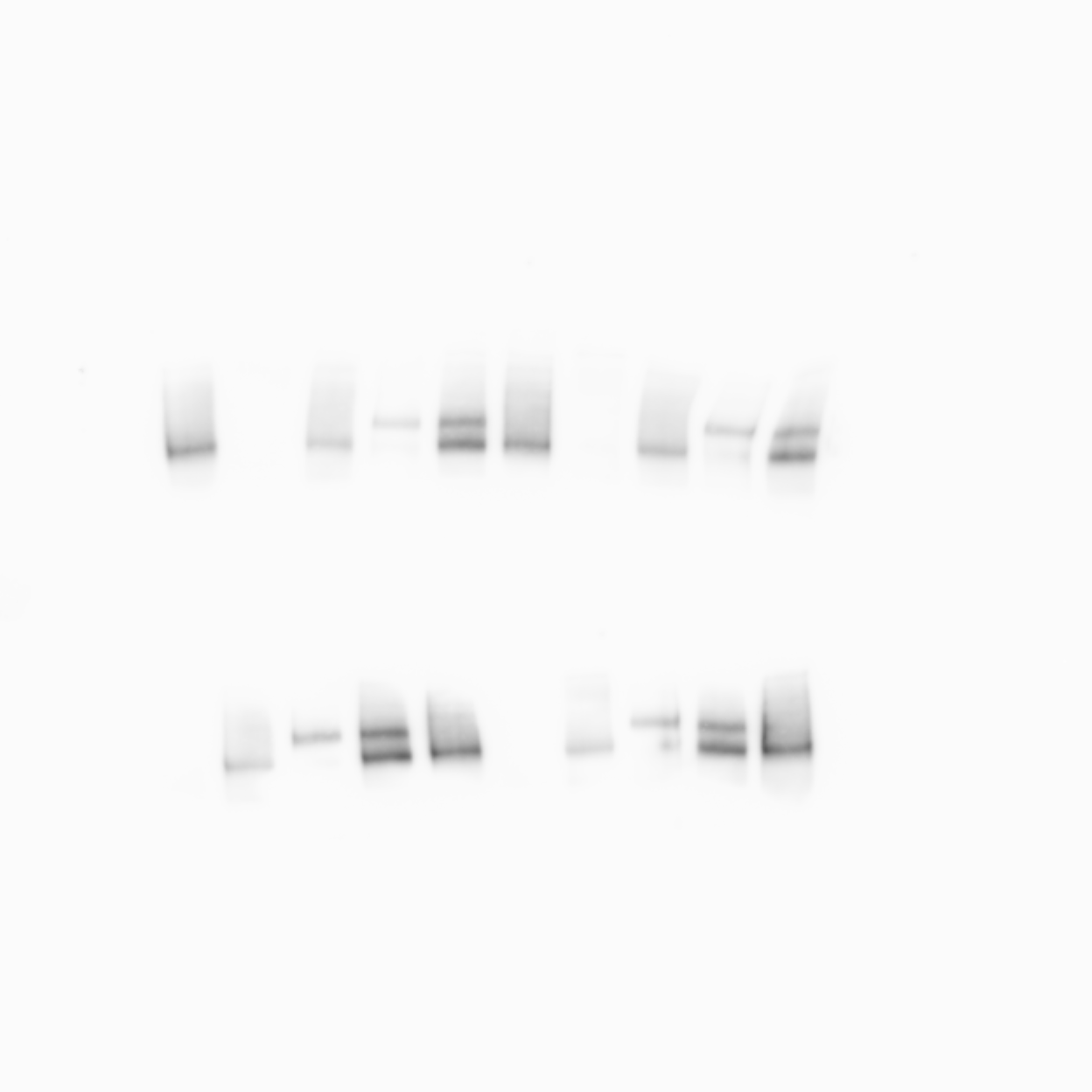

Supplement: Figure 6—figure supplement 1—source data 3. [file elife-101652-fig6-figsupp1-data3.zip › Figure 6-figure supplement 1-source data 3/Figure 6-supplement 1C-pY1173.tif]

A

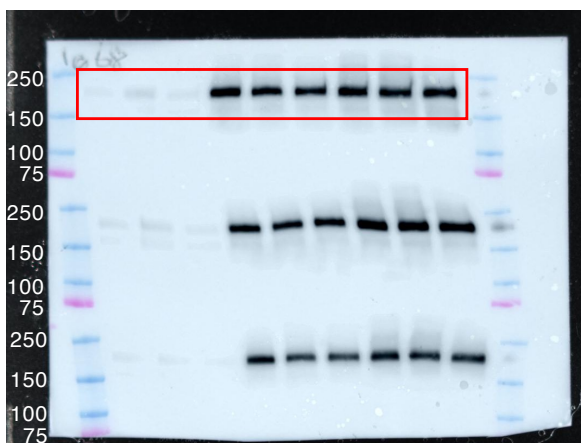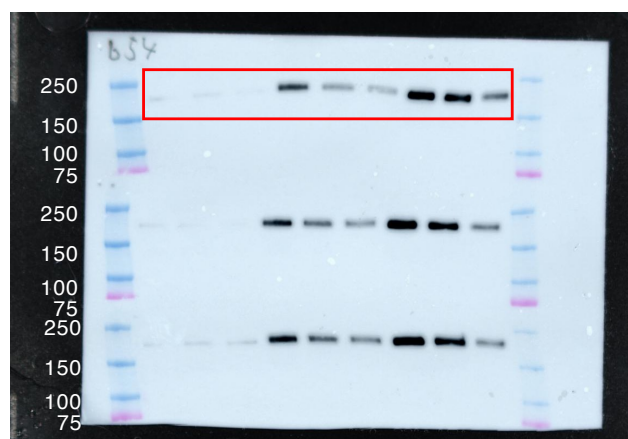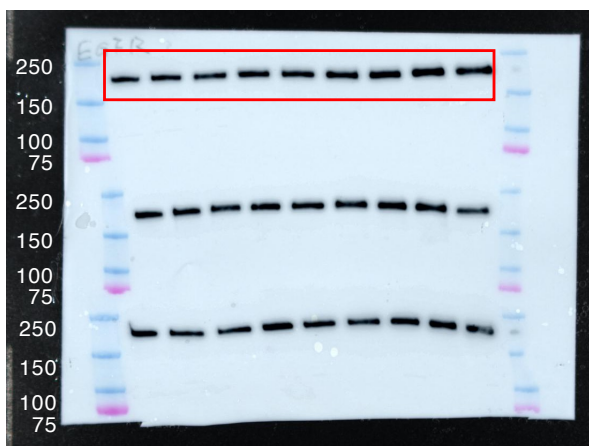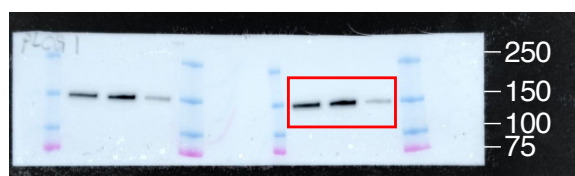

C

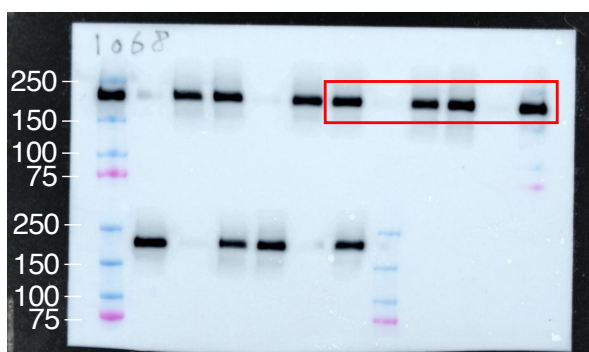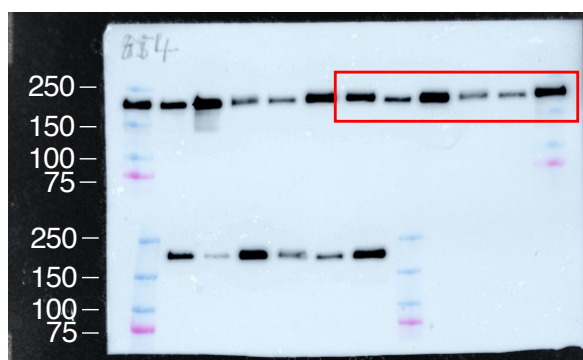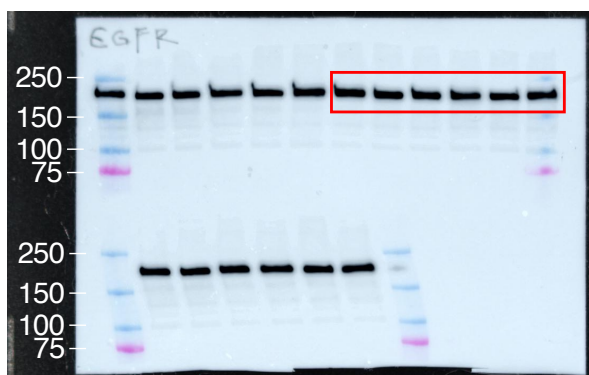

Supplement: Figure 7—source data 1. [file elife-101652-fig7-data1.zip › Figure 7-source data 1.pdf]

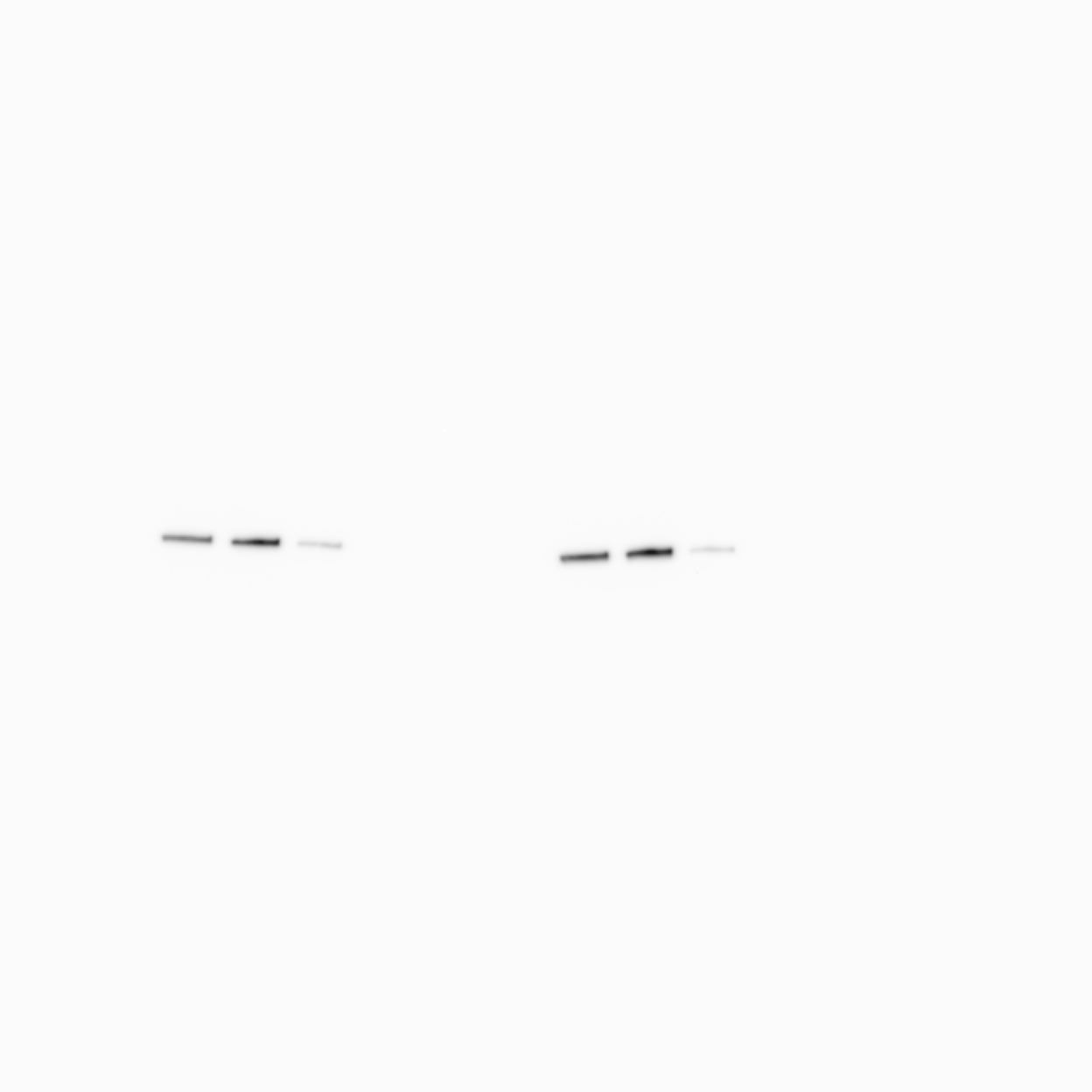

Supplement: Figure 7—source data 2. [file elife-101652-fig7-data2.zip › Figure 7-source data 2/Figure 7A-PLCG1.tif]

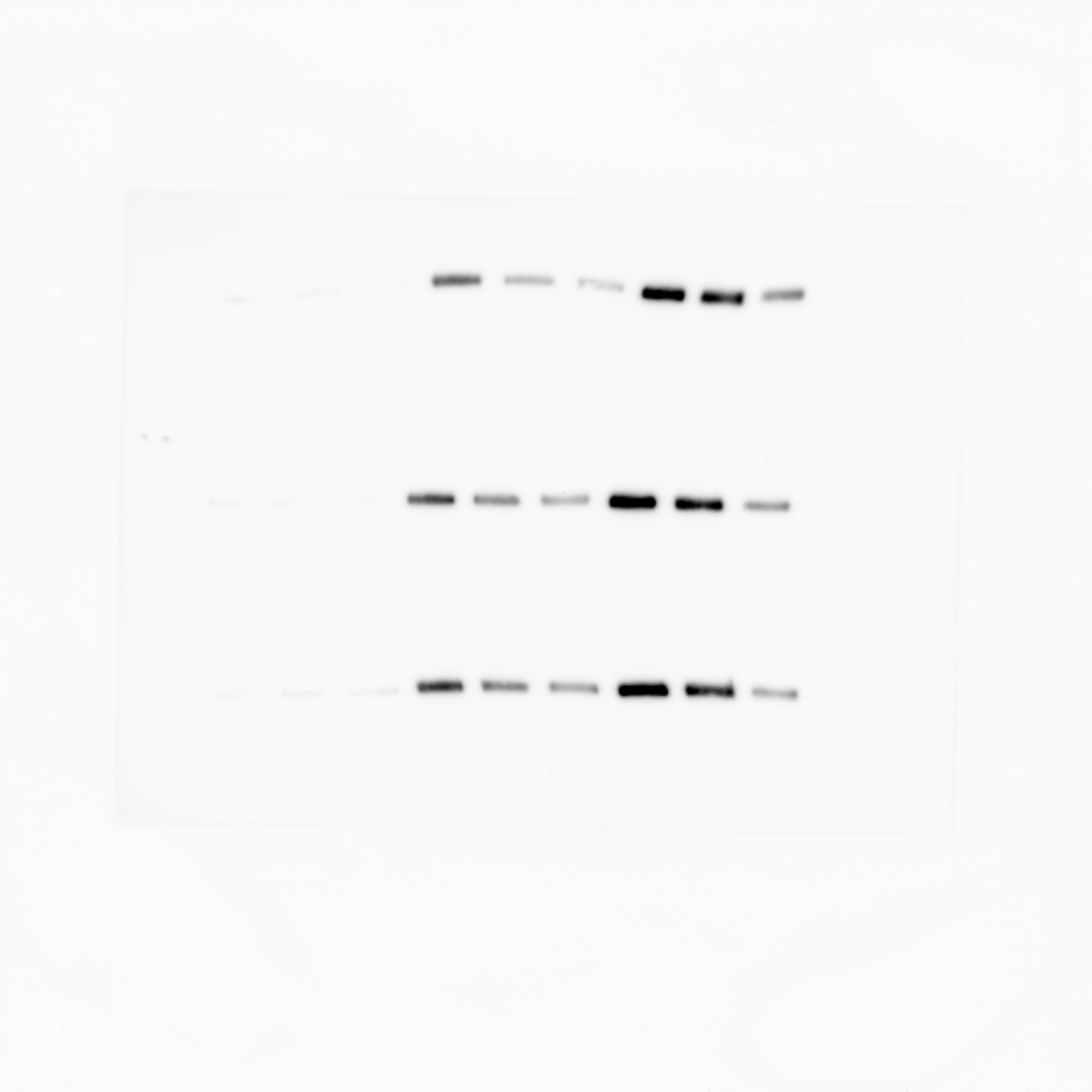

Supplement: Figure 7—source data 2. [file elife-101652-fig7-data2.zip › Figure 7-source data 2/Figure 7A-pT654.tif]

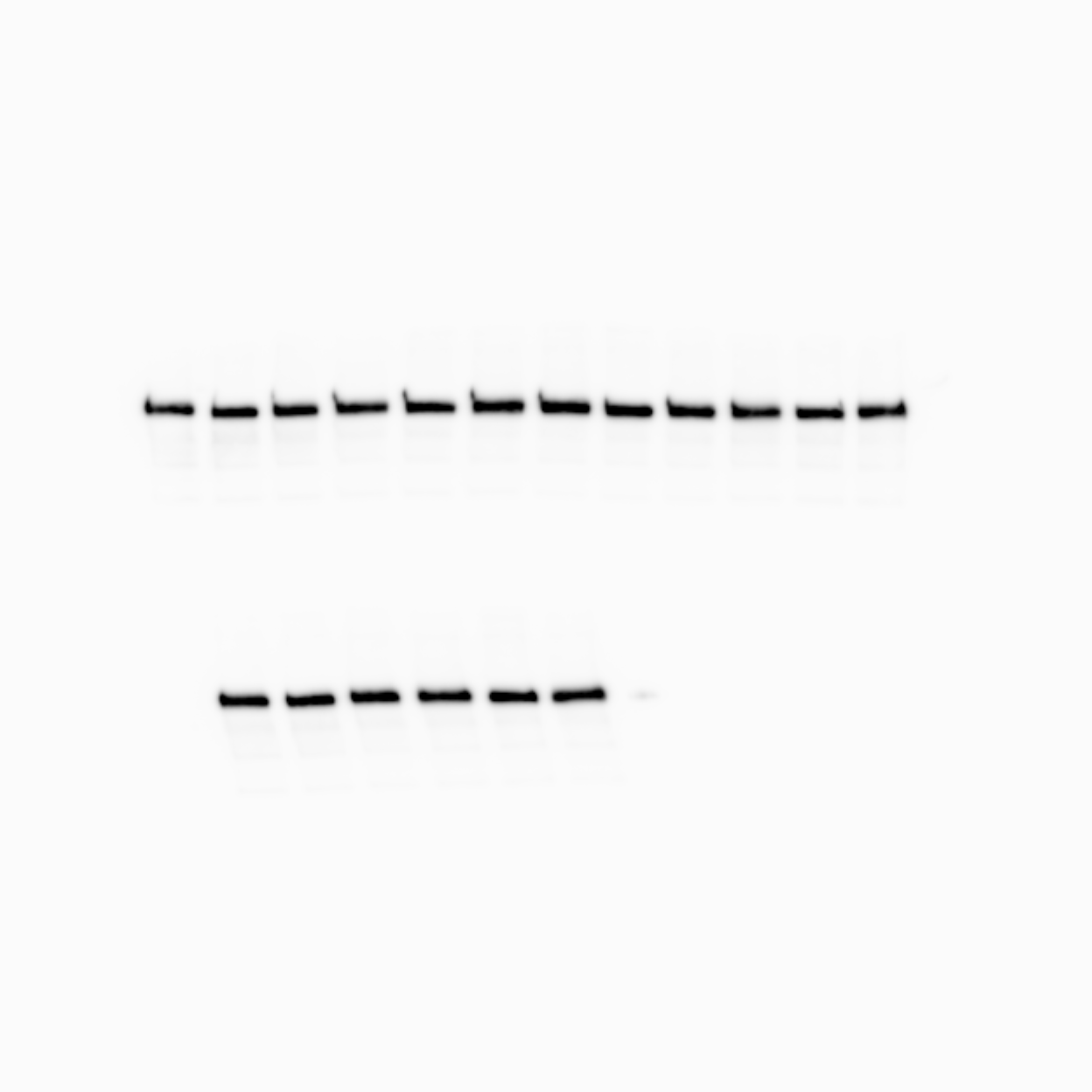

Supplement: Figure 7—source data 2. [file elife-101652-fig7-data2.zip › Figure 7-source data 2/Figure 7C-EGFR.tif]

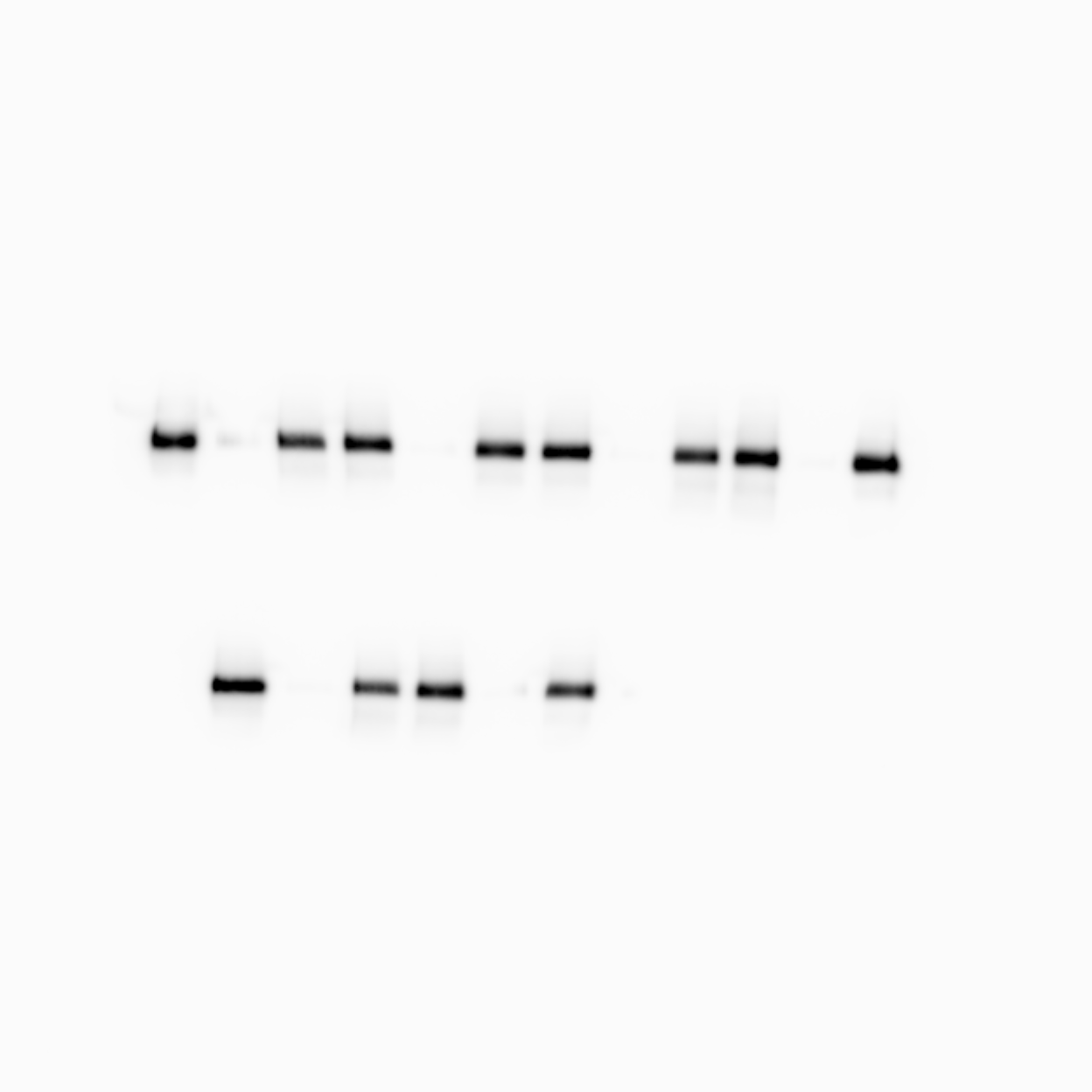

Supplement: Figure 7—source data 2. [file elife-101652-fig7-data2.zip › Figure 7-source data 2/Figure 7C-pY1068.tif]

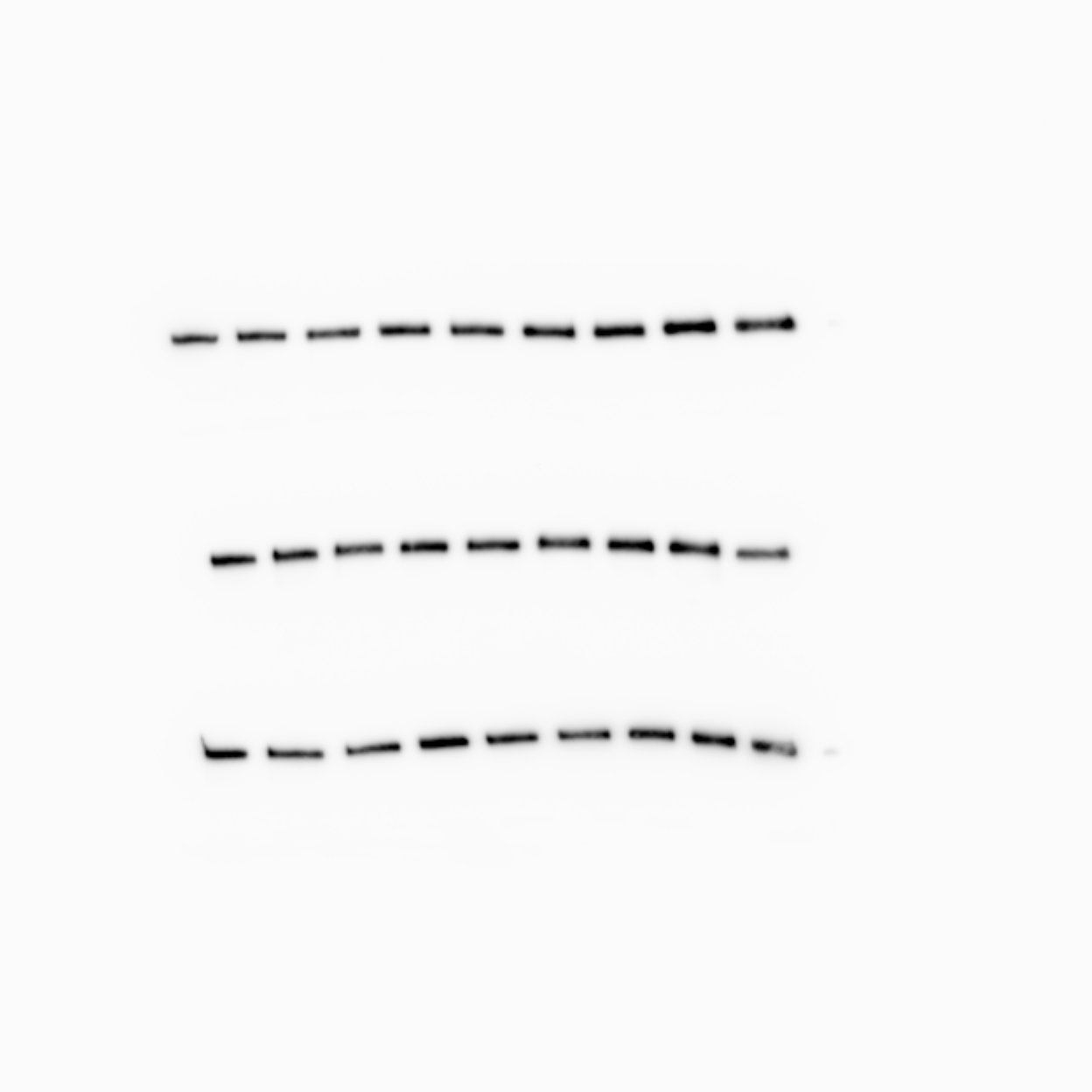

Supplement: Figure 7—source data 2. [file elife-101652-fig7-data2.zip › Figure 7-source data 2/Figure 7A-EGFR.tif]

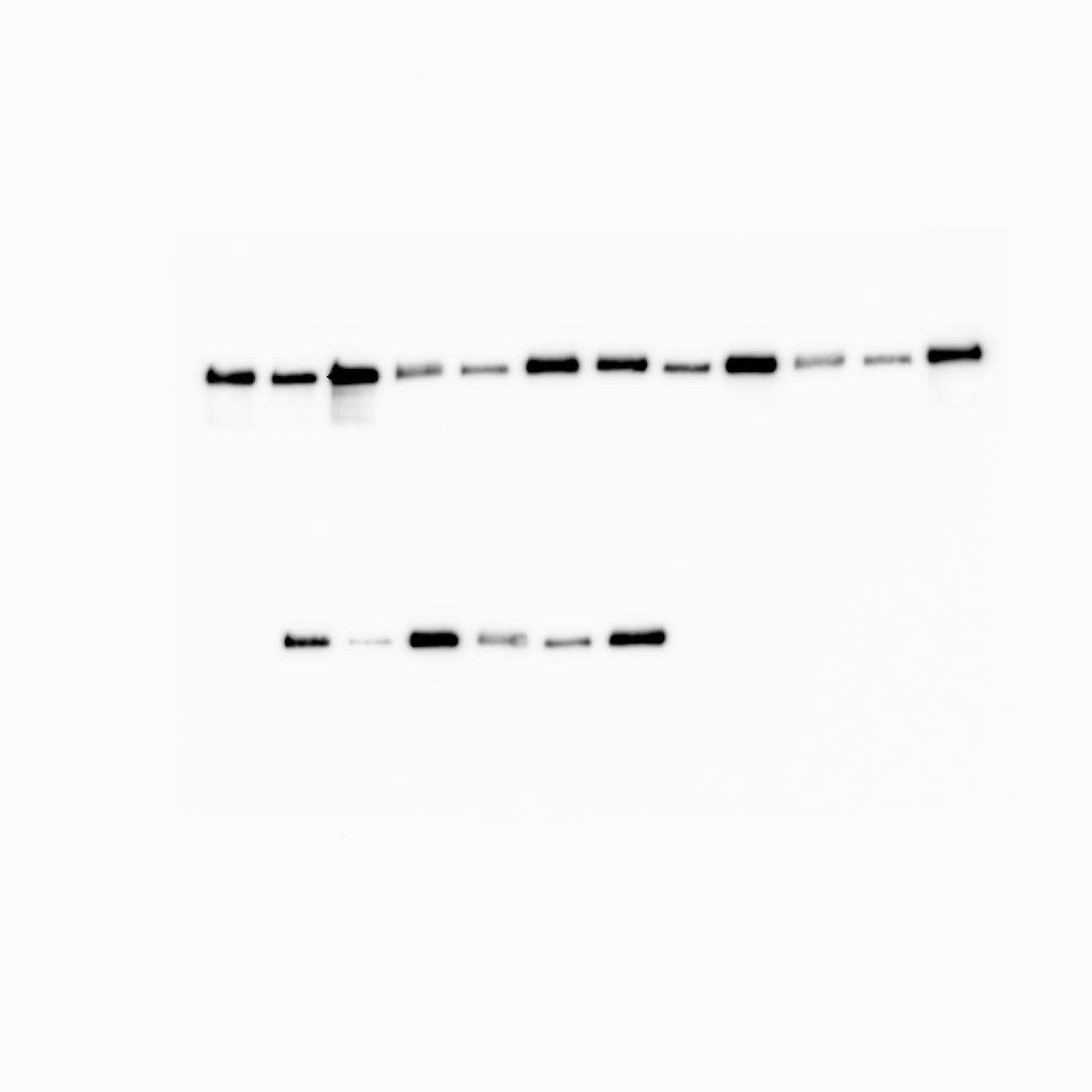

Supplement: Figure 7—source data 2. [file elife-101652-fig7-data2.zip › Figure 7-source data 2/Figure 7C-pT654.tif]

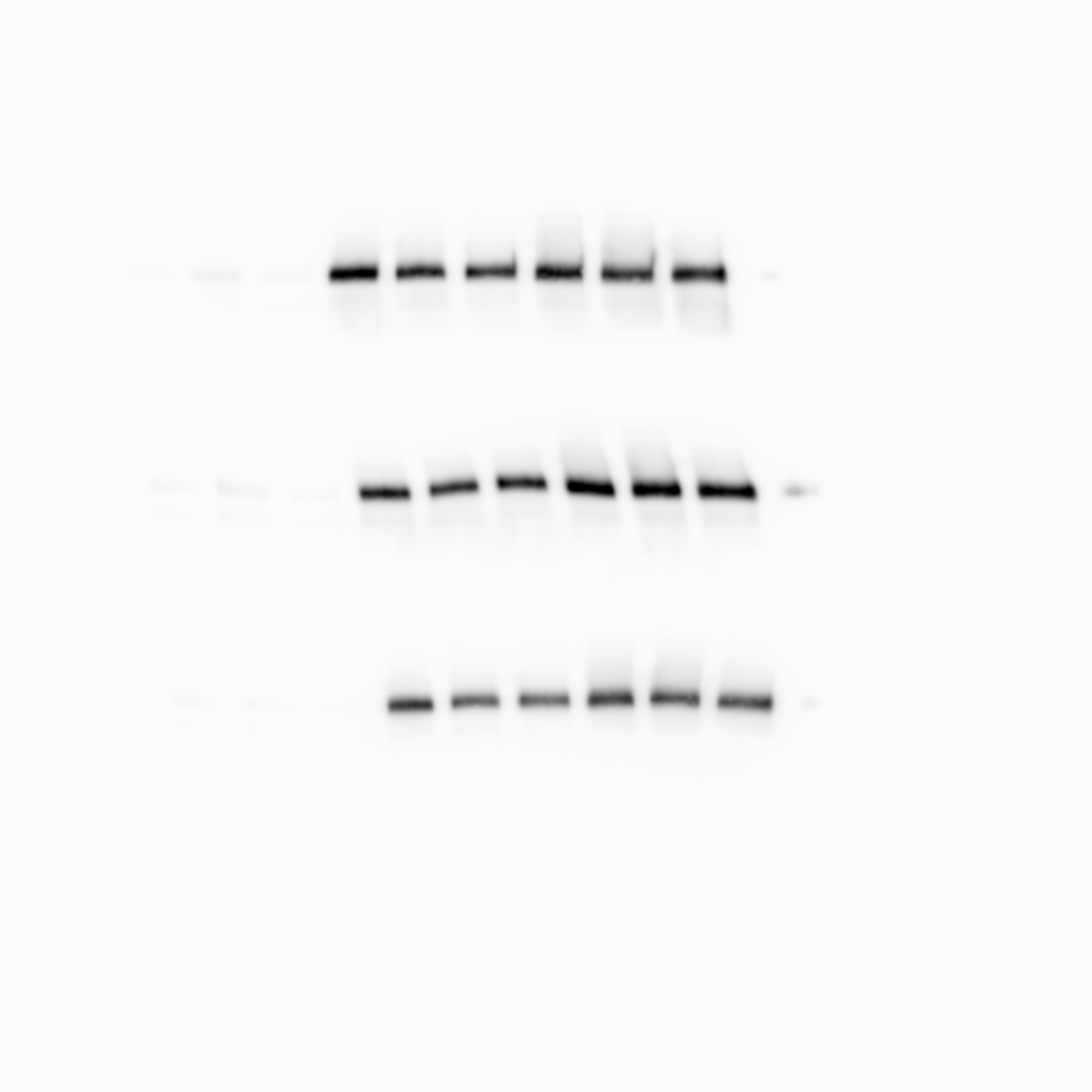

Supplement: Figure 7—source data 2. [file elife-101652-fig7-data2.zip › Figure 7-source data 2/Figure 7A-pY1068.tif]
